# Supply and demand matching of public sports services for rural older adults in China: current situation and related factors

**DOI:** 10.3389/fpubh.2025.1662362

**Published:** 2025-11-14

**Authors:** Hai-pei Zhang, Cong-hui He, Zhi-hua Wang, Wen-yun LU

**Affiliations:** 1 School of Economics and Management, Shanghai University of Sport, Shanghai, China; 2 School of Physical Education, Shanghai University of Sport, Shanghai, China; 3 College of Physical Education, Sichuan University, Chengdu, China

**Keywords:** rural older adults, public sports services, supply and demand matching, service accessibility, Chinese practice

## Abstract

**Introduction:**

China's rural aging population faces significant health challenges. This study examines the supply-demand matching of public sports services for rural older adults to promote healthy aging.

**Methods:**

We surveyed adults aged 50+ (demand side) and village cadres (supply side) across eastern, central, and western China. Data were collected via questionnaire surveys and analyzed using canonical correlation analysis (CCA).

**Results:**

Overall supply-demand matching was low, with significant deficiencies in sports funding, facilities, and activity organization. Matching levels were higher in eastern and economically developed villages. Older adults' demand correlated significantly with educational background, economic status, and health conditions. CCA revealed that service matching enhanced physical activity awareness but failed to address needs related to health status and lifestyle, showing notable gaps in satisfaction and participation.

**Discussion:**

To optimize services, we propose improved strategic planning, multi-entity collaboration, demand-oriented content, and integrated online-offline platforms to better support healthy aging in rural China.

## 1 Introduction

With the deepening of population aging in China, the older adult population in rural areas continues to expand, and health problems are becoming more and more prominent (1). For a long time, rural older adults have faced many problems, such as insufficient medical resources (2), poor health awareness (3), and sedentary lifestyles. The high incidence of chronic diseases (4) and the decline of physical functions (5) have become a common phenomenon. How to promote the physical health of rural older adults through scientific and accessible means has become an important topic in the governance of rural public health. As an important way to improve the health of older adults, physical exercise has been emphasized in more and more studies and policies. Policy documents, such as the *National Fitness Plan (2021–2025)* and the “*Healthy China 2030” Plan*, emphasize the need to accelerate the construction of a public sports service system, promote the extension of national fitness to the grassroots level, and improve the coverage of services for key groups such as older adults (6, 7). Against this backdrop, rural seniors as a group are simultaneously facing resource scarcity and health risks; their access to sports services deserves greater attention. Existing research has made preliminary explorations into the content, characteristics, and related factors of public sports needs among rural seniors. However, most studies either focus solely on demand-side analysis or merely provide descriptive accounts of the supply-side status quo. Systematic discussions on the relationship between supply and demand have yet to emerge, and there remains a lack of in-depth research combining empirical data to reveal mismatches and uncover influencing mechanisms.

In fact, the core bottleneck in rural public sports services today lies precisely in the structural imbalance between supply and demand. A significant gap exists between the personalized and diverse demands on the demand side and the standardized, homogeneous services on the supply side. This gap directly constrains the actual effectiveness of public sports services in safeguarding the health of rural seniors. Based on this, this study adopts a core perspective of supply-demand matching to fill the research gap in existing literature, which emphasizes single dimensions while neglecting systemic connections. On one hand, it overcomes the limitations of previous studies that focused solely on either demand or supply by integrating both sides into a unified analytical framework. Through field surveys, it accurately captures the genuine needs of rural seniors while comprehensively mapping the current state of public sports service provision. On the other hand, it introduces canonical correlation analysis (CCA) to quantitatively dissect the overall state of supply-demand matching and its key related factors. This provides theoretical and practical references for achieving healthy aging goals and advancing the high-quality development of rural public sports services.

## 2 Research subjects and methods

### 2.1 Research subjects

This study focuses on the matching of supply and demand of public sports services for rural older adults in China. Regarding the definition of the age boundary for older adults, the World Assembly on Aging held by the United Nations in 1982 proposed that people aged 60 and above are recognized as older adults. Foreign studies on sports for older adults have different standards, with some taking 65 years as the boundary and others taking 60 or even 50 years as the starting point (8, 9). Considering that the average life expectancy of rural older adults in China is generally about 12 years shorter than that of urban residents (10), and that around the age of 50 is the stage of high incidence of chronic diseases, in order to better promote the early intervention and guidance of physical activity behaviors. This article defines the age of rural older adults as 50 years old and above. This decision, while differing from the UN standard of 60 years, is based on two key considerations specific to the Chinese rural context. First, the average life expectancy of rural residents is notably shorter than that of urban residents, and chronic diseases often manifest earlier, around the age of 50. Second, from a public health perspective, defining the target population from age 50 aligns with a strategy of early intervention and health promotion, aiming to establish healthy habits before the onset of more severe age-related decline. We acknowledge that this tailored definition may affect the direct international comparability of our findings, but we argue it enhances the study's relevance for domestic policymaking. Therefore, the demand-side subjects of this study are people aged 50 and above living in townships, market towns, and villages corresponding to townships, while the supply-side subjects are village-level cadres responsible for cultural and sports work in the above areas.

### 2.2 Methods

#### 2.2.1 Questionnaire

Literature review, questionnaire survey, interview survey and statistical analysis were used in this study. This study used stratified sampling and simple random sampling to select samples. Questionnaires on the demand of public sports services for rural older adults were distributed, targeting rural older adults of Fuding City (Fujian Province, eastern China), Shaoyang County (Hunan Province, central China) and Guanghan City (Sichuan Province, western China). When distributing questionnaires, investigators conducted door-to-door surveys and assisted respondents in filling out the forms through interviews. A total of 1,800 questionnaires were distributed in our study. Excluding 133 invalid questionnaires, 1,667 valid questionnaires remained, with an effective recovery rate of 92.6% (invalid responses are determined based on the following criteria: ① missing key questions (matching criteria/stratification variables), ② duplicate records for the same respondent or address, ③ age < 50 or failure to meet screening criteria, ④ logical conflicts (e.g., contradictory responses to mutually exclusive questions), ⑤ abnormal responses (extremely short completion time/straight-line answering patterns). The sample showed that in terms of gender, there were 906 males (54.35%), which was slightly higher than the number of females. In terms of age, the sample consisted mainly of young older adults (under 70 years old), which accounted for 70.12% of the total sample. In terms of education, more than 70% of older adults in the sample had an education level of “primary school and below.” In terms of income, the largest group is those with “average” financial status, numbering 1,047 people (62.81%). The group with “more difficult” economic situations is relatively small, and the group with “affluent and above” families is the smallest group. The group belonging to “well-off and above” families is the smallest. In terms of marital status, the proportion of older persons with a spouse (74.09%) is much higher than that of older persons without a spouse. In terms of regional structure, the sample was relatively evenly distributed, with 437 (26.24%) in the eastern region, 507 (30.44%) in the central region and 723 (43.32%) in the western region. The sample of older adults and village cadre was merged with equal weighting at a 50/50 ratio to balance the two groups and prevent bias from sample imbalance. Robustness checks were performed on population weights (stratified by “East/Central/West × village economic tier,” using iterative quota/raking, extreme weights truncated at 1%-99%). The “achieved matching” rate and 95% confidence interval (CI) were recalculated under these weights. Results were nearly identical to the equal-weight approach (differences within ± 0.8 percentage points). It is important to note that the demographic characteristics of the demand-side sample—such as the slight male majority (54.35%), the high proportion with primary school education or below (>70%), and the predominance of younger older adults (under 70 years old, 70.12%)—are highly reflective of the current socio-demographic reality of rural China. This is largely due to long-term patterns of out-migration where more young and middle-aged women leave rural areas for urban employment, and the historical context of limited educational access for this older cohort. Therefore, while the sample may appear skewed, it accurately represents the target population for public sports services in these regions. Overall, the demographic structure of the sample is relatively consistent with the actual living status of rural older adults. The sample sizes in the eastern, central, and western regions are appropriate and even, making it highly representative.

At the same time, in order to understand the matching of supply and demand of public sports services for rural older adults in China, a questionnaire on the current situation of the supply of public sports services for rural older adults was designed, which included the matching of supply and demand of public sports services for rural older adults in China. Fuding City of Fujian in the eastern China, Shaoyang County of Hunan in the central China, and Guanghan City of Sichuan in the western China were selected, according to the degree of economic development, one town (township) each with high, medium, and low levels was selected from each city (county), and the questionnaire on the current situation of the supply of public sports services for rural older adults was distributed to all villages (communities) in the nine townships. In addition, some villages (communities) were selected from other townships in the three cities (counties) for investigation. In order to fully understand the national situation, we took the opportunity of the national “village chiefs” forum held in Baoshan village (Pengzhou, Sichuan province) to distribute the questionnaires to the cadres in charge of culture and sports work in administrative villages. A total of 587 questionnaires were collected, 25 invalid questionnaires were excluded, leaving 563 valid questionnaires, with an effective recovery rate of 95.9%. In the sample, there were 91 samples in ethnic minorities and 472 samples in non-ethnic minorities. In the ethnic minority regions, the sample sizes in the east, central, and west accounted for 25.3%, 4.4%, and 70.3%, respectively. In the non-ethnic minority regions, the sample sizes in the east, central, and west accounted for 6.4%, 8.5%, and 82.6%, respectively. As a whole, the proportions in the east, central, and west were 9.4%, 8.0%, and 82.6%, respectively.

#### 2.2.2 Confirmatory factor analysis

The questionnaire items in this study all employed a 5-point Likert-type scale. In the confirmatory factor analysis (CFA), the WLSMV estimator was primarily used. This method is based on polychoric correlation matrices and employs mean- and variance-adjusted Chi-square statistics. For comparison, robust maximum-likelihood estimation (MLR, Satorra-Bentler scaled) was also conducted. Regarding the evaluation of model fit, the reported indices include χ^2^(df), Comparative Fit Index (CFI), Tucker-Lewis Index (TLI), Root Mean Square Error of Approximation (RMSEA) (with 90% CI), and Standardized Root Mean Square Residual (SRMR). For all questionnaire items, standardized factor loadings with their corresponding 95% CIs are presented. Additionally, the composite reliability (CR), average variance extracted (AVE), and McDonald's ω for the latent constructs were calculated to comprehensively reflect the internal consistency and convergent validity of the measurement instrument.

Contingency table analysis (hypotheses, effect sizes, and multiplicity). For comparisons between groups involving categorical variables, Pearson's χ^2^ test was used, consistently reporting the degrees of freedom (df), *p*-value, and the effect size Cramér's *V* with its 95% CI (calculated using the non-central Chi-square method). For each contingency table, the minimum expected frequency was first checked: if the proportion of cells with expected count < 5 was ≥20% or if any zero counts were present, Fisher's exact test (using the Monte Carlo method with 10,000 replications) was employed instead, or adjacent categories were merged. The Benjamini-Hochberg (BH) procedure was applied to control the false discovery rate (FDR) at 5%. For significant results, the *q*-value is also reported. Unless otherwise specified, conclusions with *p* < 0.001 remained significant after FDR control, whereas results with *p* close to 0.05 were mostly non-significant after FDR adjustment.

Factor loadings reflect the relationship between an item and its underlying latent variable, not the degree of supply-demand “match.” Therefore, we avoid directly using the magnitude of factor loadings to rank the “degree of matching.” If comparison is necessary, the proportion of respondents indicating a “match” for each item in the sample, along with its 95% CI, serves as a more appropriate basis, as it aligns better with the meaning of the observed data. Furthermore, to ensure the comparability of results across different groups, we tested for measurement invariance across (eastern, central, and western) regions and village economic levels (underdeveloped, average, and developed). The testing sequence followed these steps sequentially: configural invariance → metric invariance → scalar invariance. The criteria for judging invariance were ΔCFI ≤ 0.010 and ΔRMSEA ≤ 0.015. If full scalar invariance was not achieved, partial scalar invariance was established by releasing some intercept parameter constraints before proceeding with cross-group comparisons of latent means.

#### 2.2.3 Chi-square test

In this study, the Chi-square test was used to analyze the differences in the degree of match between different ethnic groups, regions, and villages with different financial status in terms of the supply and demand of public sports services for rural older adults (including investment in sports, construction of venues and facilities, organizational construction, training of backbones, organization of sports activities, fitness guidance, provision of health knowledge, publicity and mobilization, system construction and physical fitness monitoring service). Whether there is a statistically significant difference between the variables in each group was determined by calculating the degree of deviation between the observed frequencies and the theoretical frequencies. The formula for calculating the Chi-square statistic is as follows:


$$\begin{array}{l} X^{2}=\sum \frac{{\left(O_{ij}-E_{ij}\right)}^{2}}{E_{ij}} \end{array}$$

where, *O*_*ij*_ denotes the observed frequency in row *i*, column *j*, and *E*_*ij*_ is the theoretical frequency of the cell, which is calculated as follows:


$$\begin{array}{l} E_{ij}=\frac{R_{i}\times C_{j}}{N} \end{array}$$

where, *R*_*i*_ is the marginal total of row *i*, *C*_*j*_ is the marginal total of column *j*, and *N* is the total number of samples. If *p* < 0.05, H_**0**_ will be rejected. If *p* > 0.05, H_0_ will be accepted.

#### 2.2.4 Canonical correlation analysis

Canonical correlation analysis is a statistical analysis method to study the interrelationship between two groups of variables. This study analyzes the correlation between the supply and demand of public sports services for rural older adults and their various related factors with the help of this method. The method adopts the principle of principal component analysis to extract components, and each of the two groups of variables is linearly combined into canonical variables. The original correlation between the two groups of variables is transformed into the study of canonical correlation between a few canonical variables proposed from each group, thus reducing the number of variables in the study. In practical application, several pairs of canonical variables are retained based on the significance test of the canonical correlation coefficient and the amount of information contained in the canonical variables. The mathematical principle of the CCA can be expressed as follows:

Let the random vector X = (x1, x2, …, xp), Y = (y1, y2, …, yq), the variance matrix of X, Y is as follows:


$$\begin{array}{l} Cov=:\left[\begin{array}{c} X\\ Y \end{array}\right]\sum =\left[\begin{array}{cc} \sum 11 & \sum 12\\ \sum 21 & \sum 22 \end{array}\right] \end{array}$$

where ∑11 is the covariance matrix of the first group of variables, ∑12 and ∑21 are the covariance matrices of the first and second group of variables, and ∑22 is the covariance matrix of the second group of variables. In order to study the canonical correlation between two groups of variables, X and Y, a linear combination between them is made as follows:


$$\{\begin{array}{c} U=a^{\prime }X=a_{1}x_{1}+a_{2}x_{2}+\ldots +a_{p}x_{p}\\ V=b^{\prime }Y=b_{1}y_{1}+b_{2}y_{2}+\ldots +b_{p}y_{p} \end{array}$$

Given x, y, and ∑ that is to say, a, b, to maximize the correlation coefficients between U and V:


$$\begin{array}{l} r=\;\frac{cov\left(U,V\right)}{\sqrt{var\left(U\right)var\left(V\right)}}. \end{array}$$

This study addressed measurement quality assessment and the examination of variable relationships separately. First, measurement model analysis was conducted using AMOS 26.0. The model was estimated with the Weighted Least Squares Mean and Variance adjusted (WLSMV)/MLR estimators. Model fit was reported using the following fit indices: χ^2^(df), CFI, TLI, RMSEA (with 90% CI), and SRMR. For all questionnaire items, standardized factor loadings with their corresponding 95% CIs are provided. Additionally, CR, AVE, and McDonald's ω were calculated for the latent constructs. To enable cross-group comparisons, measurement invariance across regions (east/central/west) and village economic level (underdeveloped/average/developed) was tested sequentially for configural, metric, and scalar invariance. The criteria for judging invariance were ΔCFI ≤ 0.010 and ΔRMSEA ≤ 0.015. Partial measurement invariance was established if full invariance was not met. Subsequently, CCA was performed using SPSS/R. Supply-side variables were designated as set X, and demand/matching variables as set Y. The analysis was based on the (polychoric) correlation matrix, proceeding only after assumptions of linearity, multicollinearity, and the absence of significant outliers were checked. For each canonical function, the following were reported: the canonical correlation (Rc), Wilks' Lambda (Λ), degrees of freedom (df), Rao's *F*, and *p*-value. Additionally, standardized canonical coefficients, structure coefficients (canonical loadings), cross-loadings, and redundancy indices (for both X → Y and Y → X) were examined. To prevent misinterpretation, the magnitude of factor loadings or canonical loadings was not used to rank the “degree of matching.” Where comparisons were necessary, only the observed proportion of “match achieved” for each item, along with its 95% CI, was provided. This analytical approach elucidated the primary correlational pathways and structural relationships between the supply and demand of public sports services for rural older adults and their various influencing factors. It thereby provides statistical support and a theoretical foundation for subsequent analysis of the allocation efficiency and intervention pathways for sports service resources for this demographic.

## 3 Concept definition

### 3.1 Public sports service for rural older adults

Defining “public sports services for rural older adults” is the starting point of this study. To clarify the connotation of “public sports services for rural older adults,” it is necessary to first clarify the basic concept of “public sports services.” Since the “public service” was first mentioned in the government work report in 2002, academic attention to public sports services has gradually increased. However, there have been two different ways of expressing “sports public service” and “public sports service” in the academic world. Scholars who support the expression “sports public service” believe that this expression is more in line with the logic and precision of the language structure, and can effectively avoid misunderstanding of the concept of “public sports” (11). Scholars in favor of the use of “public sports service” believe that “public sports service” is consistent with the expression habits of other public utility fields, such as “public education service,” “public healthcare service,” etc., which is more uniform and standardized (12). In addition, from the perspective of terminological structure, word attributes, language habits and practical application, the expression “public sports service” is more reasonable and appropriate (13). Although the choice of the two terms has caused some controversy in the early stages of research, in fact, the two expressions refer to the same thing, and there is no substantial difference. In this study, we prefer to use the term “public sports service” to be consistent with the terminology used in official policy documents such as the *14th Five-Year Plan for Sports Development*, the *National Fitness Plan (2021–2025)*, and the *Outline for Building a Leading Sports Nation*.

Public sports service is an important part of China's “*14th Five-Year” Plan for public services*. Together with education, housing security, healthcare, and other areas, it constitutes the basic public service system in China. At present, the definition of “public sports services” in the academic world shows a diversified trend. From the perspective of public goods theory, public sports service is a public product that meets the needs of the general public and has the qualities of non-exclusivity and non-competitiveness (14). From the perspective of organizational function, public sports service emphasizes the service function of meeting the public's needs for sports (15). And from the perspective of public interest, public sports service aims to realize the overall interests of society, relying on the government's resources and power to provide the corresponding services or activities (16). At the level of service providers, some studies point out that sports services provided by the government, enterprises or third-party organizations are public sports services as long as they serve the public interest (13). This study prefers to adopt the public interest perspective, arguing that the core of public sports services lies in the realization and manifestation of public interests, and that publicness must be reflected through the realization of public interests. With the report of the 19th CPC National Congress pointing out that socialism with Chinese characteristics has entered a new era, the connotation of public sports services has been continuously expanded and deepened. How to meet the diversified and multi-level sports and fitness needs of the people and ensure the fair realization of the right to participate in sports has become the core task and mission of public sports services in the new era. Therefore, this study defines “public sports service” as the various behaviors or activities with tangible and intangible objects as the carrier that are carried out by government-led pluralistic subjects, in order to achieve the public interests of sports, using their respective resource advantages, and adhering to the practical principle of people-centered.

Based on the above understanding, rural older adults covered by this study specifically refer to older adults aged 50 and above living in townships, market towns, and villages corresponding to townships. Accordingly, this study clearly defines “public sports services for rural older adults” as various behaviors or activities with tangible and intangible objects as the carrier that carried out by government-led pluralistic subjects, in order to achieve the public interest in sports, using their respective resource advantages, and targeting people aged 50 and above living in townships, market towns, and villages corresponding to townships, and adhering to the practical principle of people-centered. Its ultimate aim is to meet the growing, diversified, and multi-level physical fitness needs of rural older adults. In this concept, the various behaviors or activities provided mainly refer to the introduction of sports policies and regulations, investment in sports funding input, construction of sports venues and facilities, organization of sports activities for older adults, monitoring of physical fitness, establishment of sound sports organizations for older adults, training and giving full play to the roles of sports cadres, and the provision of sports information and consulting services.

### 3.2 Matching of supply and demand of public sports services for rural older adults

In order to better understand what is meant by “matching of supply and demand of public sports services for rural older adults,” it is necessary to clarify the connotations of “demand” and “supply” and their interrelationships first. From the demand side, the demand of public sports services for rural older adults refers to the subjective will and objective needs of rural older adults for sports services in a specific social environment and health background. Specifically, this demand is reflected in rural older adults' expectation for public sports services, such as sports facilities, fitness activities, professional guidance, organizational support, etc., based on their own health status, lifestyle, sports knowledge and other factors within a certain period of time. When there is a gap between their expectations and the reality of available sports service resources, the demand state of “lack of service” is formed (17). From the supply side, the supply of public sports services for rural older adults refers to the dynamic process in which the government takes the lead and social organizations and market entities participate jointly to provide public welfare, age-friendly, and sustainable sports services for rural older adults through financial investment, policy guidance, staffing, and facility construction. The core of the process lies in the continuous improvement of the institutional system and service mechanism, the enhancement of accessibility, professionalism and fairness, and the realization of the universality and precision of public sports services.

On this basis, the matching of supply and demand of public sports services for rural older adults is essentially a multi-dimensional and multi-factor dynamic coordination process. It aims to optimize the structure of resource allocation and improve the adaptability of service supply by accurately identifying the diverse demands for sports service among older adults, so as to achieve the matching of supply content and demand preferences, the matching of supply capacity and service object characteristics, and the integration of supply mode and the acceptance habits of older adults. Only by promoting the dynamic coupling of supply and demand through multiple paths, such as systems, resources, personnel and technology, can we effectively improve the quality of services and the participation rate of older adults, enhance the responsiveness and resilience of the public sports service system, and truly realize the service pattern of “demand-determined supply, supply-demand interaction, and accurate supply.” In previous studies, the indicators for determining the matching of supply and demand of public sports services for older adults mainly include sports venues and facilities, sports and fitness guidance, sports organizations, sports activities, sports health knowledge, and physical fitness monitoring and other aspects (18). This study combined the actual situation selected 10 representative indicators, as shown below.

### 3.3 Matching baseline

This study defined “achieved match” as the aggregate of the response categories “basic match,” “comparative match,” and “complete match.” The definition of the matching baseline referenced the standardized “baseline/floor” criterion used for assessing compliance in basic public services, as stipulated in the 2024 national standard “*Evaluation Principle of Basic Public Services Equalization”* in China (19). It also aligned with the threshold mentioned in documents from the Ministry of Finance (20) and local performance guidelines (21), where a value below 60% is considered a failing score or indicative of non-compliance. Consequently, this study defined a “matching baseline” below 60% as indicative of a significant supply-demand problem.

## 4 Results and analyses

### 4.1 Analysis of the degree of matching of specific indicators of the supply and demand of public sports services for rural older adults

In order to comprehensively analyze the problem of matching of supply and demand of public sports services for rural older adults, find out the differences in regions, ethnic groups and economic development levels of the mismatch, and accordingly reveal the influence mechanism of the matching supply-demand relationship on the physical activity of older adults, it is necessary to prioritize the understanding of the current evaluation of the village leaders on the matching of the supply and demand of public sports services for older adults, and to verify the validity and reliability of the measurements. This study designed a total of 10 questions: ① investment in sports for older adults, ② construction of sports venues and facilities for older adults, ③ construction of sports organizations for older adults, ④ training of backbone of sports for older adults, ⑤ organization of sports activities for older adults, ⑥ guidance for older adults participating in sports activities, ⑦ provision of health and fitness knowledge for older adults, ⑧ publicity and mobilization for older adults to participate in sports, ⑨ system construction of sports for older adults, and ⑩ physical fitness monitoring service for older adults. The degree of matching of supply and demand for each content is classified as “complete match,” “comparative match,” “basic match,” “mismatch,” and “complete mismatch,” and assigned 5, 4, 3, 2, and 1 points, respectively. The overall score of the matching of supply and demand ranges from 10 to 50 points. The higher the score, the more the supply-demand relationship.

**Figure 1:**
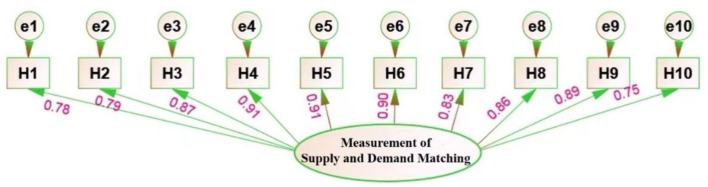
Verification of a model for measuring the matching of supply and demand for public sports services for older adults.

To avoid relying solely on point estimates, this study reports the weighted proportion of “achieved match” for each indicator along with its 95% CI (calculated using the Wilson score method, and supplemented with the BCa bootstrap method, *B* = 2,000, for small samples or uneven weighting). Sensitivity judgments are provided under the two threshold sets (55%/65% and 50%/70%). A result is defined as “borderline” if the point estimate is within ≤ 2 percentage points of a threshold and its 95% CI crosses that threshold. Rankings and comparisons are based solely on the observed proportions, not on factor loadings. CFA was conducted on the matching of supply and demand of public sports services for rural older adults. The results showed that (as shown in Figure 1) the RMSEA of the measurement model was 0.016 (< 0.05), and its goodness-of-fit index CFI, AGFI, and RFI were 0.993, 0.978, and 0.992, respectively, all exceeding the standard of 0.90, which indicates that the model is well-fitted to the data obtained from the research, indicating that the scale has good measurement validity.

Regarding the standardized coefficients (β) of the measurement model, the values for H1 through H10 are 0.78, 0.79, 0.87, 0.91, 0.91, 0.90, 0.83, 0.86, 0.89, and 0.75, respectively. These results indicate that the corresponding variance explained (β^2^) by each factor is 60.84%, 62.41%, 75.69%, 82.81%, 82.81%, 81.00%, 68.89%, 73.96%, 79.21%, and 56.25%, respectively. It can be seen that the ranking of the degree of matching of supply and demand of the 10 major content systems of public sports services for rural older adults, from the best to the worst, is as follows: “training of backbone of sports for older adults” and “organization of sports activities for older adults” are tied for first place. The third is “guidance for older adults participating in sports activities.” The fourth is “system construction of sports for older adults.” The fifth is “construction of sports organizations for older adults.” The sixth is “publicity and mobilization for older adults to participate in sports.” The seventh is “provision of health and fitness knowledge for older adults.” The eighth is “construction of sports venues and facilities for older adults.” The ninth is “investment in sports for older adults.” The 10th is “physical fitness monitoring service for older adults.”

The differing factor loadings offer practical insights. The high loadings for “training of the backbone of sports for older adults” (β = 0.91) and “organization of sports activities for older adults” (β = 0.91) suggest these are perceived by village leaders as core, well-defined components of public sports services. In contrast, the lower loading for “physical fitness monitoring service for older adults” (β = 0.75) may reflect its conceptual ambiguity in rural settings, where it is often confused with general medical check-ups, leading to a less distinct and consistent evaluation of its supply-demand matching.

### 4.2 Analysis of differences in the degree of matching of supply and demand of public sports services for rural older adults

In order to have a more concise and clearer explanation of the problem of the matching of supply and demand of public sports services for rural older adults, and in view of the fact that public sports services for rural older adults in China are still in the stage of development from scratch, this study focuses on the overall matching situation. Therefore, the village leaders' evaluations of the problem were integrated first, and the percentages of “basic match,” “comparative match” and “complete match” responses were summed up, which is called the “matching baseline.” If the value of “matching baseline” is less than 60%, it indicates that there is a problem in the supply-demand relationship.

#### 4.2.1 Analysis of differences in the matching degree of the investment in sports for older adults

As shown in Table 1:

(1) The overall assessment of the matching degree of the investment in sports for rural older adults is that 23.8% of the respondents think it is “basic match,” 13.3% think it is “comparative match,” and 6.8% think it is “complete match.” The value of “matching baseline” is 43.9%, failing to meet the standard.
(2) There was no significant difference in the matching degree of the investment in sports for rural older adults between villages of different ethnic groups (*X*^2^ = 8.485, *p* = 0.075 > 0.05), but there were significant differences between villages of different regions and levels of economic development (*X*^2^ = 55.74, *p* = 0.000 < 0.01 and *X*^2^ = 60.12, *p* = 0.000 < 0.01, respectively). In-depth analysis revealed that the values of “matching baseline” between different regions were highest in the eastern region (83.0%), followed by the western region (42.0%), and the lowest in the central region (17.8%). From the perspective of the economic conditions of the villages, the value of “matching baseline” of the matching degree of sports funding input in the “relatively developed” villages was significantly higher than that in the “general” villages, and that in the “general” villages was significantly higher than that in the “underdeveloped” villages (corresponding matching baseline: 63.5% vs. 56.7% vs. 31.6%).

**Table 1:** Analysis of difference in the matching degree of the investment in sports for rural older adults among different villages.

**(*N* = 541)**	**Complete mismatch**	**Mismatch**	**Basic match**	**Comparative match**	**Complete match**
**Different ethnic groups**					
Ethnic minorities	20 (25.3)	20 (22.0)	22 (24.2)	15 (6.5)	11 (12.1)
Non-ethnic minorities	109 (24.2)	151 (33.6)	107 (23.8)	57 (12.7)	26 (5.8)
Test	*X*^2^ = 8.485, *p* = 0.075				
**Different regions**					
Eastern	3 (5.7)	6 (11.3)	24 (45.3)	13 (24.5)	7 (13.2)
Central	23 (51.1)	14 (31.1)	3 (6.7)	2 (4.4)	3 (6.7)
Western	106 (23.9)	151 (34.1)	102 (23.0)	57 (12.9)	27 (6.1)
Test	*X*^2^ = 55.74, *p* < 0.001^***^				
**Different levels of economic development**					
Underdeveloped	102 (34.3)	101 (34.0)	49 (16.5)	28 (9.4)	17 (5.7)
General	16 (10.1)	53 (33.3)	54 (34.0)	23 (14.5)	13 (8.2)
Relatively developed villages	14 (16.5)	17 (20.0)	26 (30.6)	21 (24.7)	7 (8.2)
Test	*X*^2^ = 60.12, *p* < 0.001^***^				
**Aggregate**	132 (24.4)	171 (31.6)	129 (23.8)	72 (13.3)	37 (6.8)

^*^p < 0.05, ^**^p < 0.01, ^***^p < 0.001.

#### 4.2.2 Analysis of differences in the matching degree of the construction of sports venues and facilities for older adults

As shown in Table 2:

(1) The overall assessment of the matching degree of the construction of sports venues and facilities for older adults is that 26.3% of the respondents think it is “basic match,” 15.6% think it is “comparative match,” and 10.6% think it is “complete match.” The value of “matching baseline” is 52.5%, failing to meet the standard.
(2) There were significant differences in the matching degree of the construction of sports venues and facilities for older adults among villages with different ethnic groups, different regions and different levels of economic development (*X*^2^ = 10.069, *p* = 0.039 < 0.05; *X*^2^ = 32.676, *p* = 0.000 < 0.01; *X*^2^ = 73.376, *p* = 0.000 < 0.01). The value of “matching baseline” was significantly higher in ethnic minority villages (56.1%) than in non-ethnic minority villages (51.6%). The value of “matching baseline” in villages in the eastern region (75.4%) was significantly higher than that in the western region (52.6%), while that in the western region was significantly higher than that in the central region (22.2%). The value of “matching baseline” was significantly higher in the “relatively developed” villages (76.4%) than that in the “general” villages (65.3%), while that in the “general” villages was significantly higher than that in the “underdeveloped” villages (38.4%).

**Table 2:** Analysis of difference in the matching degree of the construction of sports venues and facilities among different villages.

**(*N* = 540)**	**Complete mismatch**	**Mismatch**	**Basic match**	**Comparative match**	**Complete match**
**Different ethnic groups**					
Ethnic minorities	20 (22.0)	20 (22.0)	17 (18.7)	22 (24.2)	12 (13.2)
Non-ethnic minorities	86 (19.2)	131 (29.2)	125 (27.8)	62 (13.8)	45 (10.0)
Test	*X*^2^ = 10.069, *p* = 0.039^*^				
**Different regions**					
Eastern	2 (3.8)	11 (20.8)	21 (39.6)	12 (22.6)	7 (13.2)
Central	18 (40.0)	17 (37.8)	5 (11.1)	2 (4.4)	3 (6.7)
Western	86 (19.5)	123 (27.8)	116 (26.2)	70 (15.8)	47 (10.6)
Test	*X*^2^ = 32.676, *p* < 0.001^***^				
**Different levels of economic development**					
Underdeveloped	87 (29.4)	95 (32.1)	59 (19.9)	30 (10.1)	25 (8.4)
General	11 (6.9)	44 (27.7)	50 (31.4)	29 (18.2)	25 (15.7)
Relatively developed villages	8 (9.4)	12 (14.1)	33 (38.8)	25 (29.4)	7 (8.2)
Test	*X*^2^ = 73.376, *p* < 0.001^***^				
**Aggregate**	106 (19.6)	151 (28.0)	142 (26.3)	84 (15.6)	57(10.6)

^*^p < 0.05, ^***^p < 0.001.

#### 4.2.3 Analysis of differences in the matching degree of the construction of sports organizations for older adults

As shown in Table 3:

(1) The overall assessment of the matching degree of the construction of sports organizations for older adults is that 26.8% of the respondents think it is “basic match,” 11.6% think it is “comparative match,” and 8.3% think it is “complete match.” The value of “matching baseline” is 46.7%, failing to meet the standard.
(2) There was no significant difference in the matching degree of the construction of sports organizations for older adults between villages of different ethnic groups (*X*^2^ = 9.065, *p* = 0.059 > 0.05), but there were significant differences between villages of different regions and levels of economic development (*X*^2^ = 33.512, *p* = 0.000 < 0.01; *X*^2^ = 48.554, *p* = 0.000 < 0.01). Specifically, the value of “matching baseline” was significantly higher in villages in the eastern region (73.6%) than that in the western region (45.4%), while that in the western region was significantly higher than that in the central region (28.9%). The value of “matching baseline” in the “relatively developed” villages (65.9%) was significantly higher than that in the “general” villages (57.2%), while that in the “general” villages was significantly higher than that in the “underdeveloped” villages (35.7%).

**Table 3:** Analysis of difference in the matching degree of the construction of sports organizations for older adults among different villages.

**(*N* = 541)**	**Complete mismatch**	**Mismatch**	**Basic match**	**Comparative match**	**Complete match**
**Different ethnic groups**					
Ethnic minorities	24 (26.4)	17 (18.7)	28 (30.8)	10 (11.0)	12 (13.2)
Non-ethnic minorities	101 (22.4)	146 (32.4)	117 (26.0)	53 (11.8)	33 (7.3)
Test	*X*^2^ = 9.065, *p* = 0.059				
**Different regions**					
Eastern	3 (5.7)	11 (20.8)	26 (49.1)	5 (9.4)	8 (15.1)
Central	18 (40.0)	14 (31.1)	8 (17.8)	1 (2.2)	4 (8.9)
Western	104 (23.5)	138 (31.2)	111 (25.1)	57 (12.9)	33 (7.4)
Test	*X*^2^ = 33.512, *p* < 0.001^***^				
**Different levels of economic development**					
Underdeveloped	93 (31.3)	98 (33.0)	60 (20.2)	24 (8.1)	22 (7.4)
General	20 (12.6)	48 (30.2)	58 (36.5)	19 (11.9)	14 (8.8)
Relatively developed villages	12 (14.1)	17 (20.0)	27 (31.8)	20 (23.5)	9 (10.6)
Test	*X*^2^ = 48.554, *p* < 0.001^***^				
**Aggregate**	125 (23.1)	163 (30.1)	145 (26.8)	63 (11.6)	45 (8.3)

^*^p < 0.05, ^**^p < 0.01, ^***^p < 0.001.

#### 4.2.4 Analysis of differences in the matching degree of the training of the backbone of sports for older adults

As shown in Table 4:

(1) The overall assessment of the matching degree of the training of the backbone of sports for older adults is that 24.4% of the respondents think it is “basic match,” 10.7% think it is “comparative match,” and 8.3% think it is “complete match.” The value of “matching baseline” is 43.4%, failing to meet the standard.
(2) There was no significant difference in the matching degree of the training of the backbone of sports for older adults between villages of different ethnic groups (*X*^2^ = 8.905, *p* = 0.064 > 0.05), but there were significant differences between different regions and levels of economic development (*X*^2^ = 40.199, *p* = 0.000 < 0.01; *X*^2^ = 48.554, *p* = 0.000 < 0.01). Specifically, the value of “matching baseline” of villages in the eastern region (71.8%) was significantly higher than that in the western region (42.2%), while that in the western region was significantly higher than that in the central region (22.2%). The value of “matching baseline” in the “relatively developed” villages (58.8%) was significantly higher than that in the “general” villages (51.6%), while that in the “general” villages was significantly higher than that in the “underdeveloped” villages (34.7%).

**Table 4:** Analysis of difference in the matching degree of the training of the backbone of sports for older adults among different villages.

**(*N* = 541)**	**Complete mismatch**	**Mismatch**	**Basic match**	**Comparative match**	**Complete match**
**Different ethnic groups**					
Ethnic minorities	26 (28.6)	19 (20.9)	21 (23.1)	14 (15.4)	11 (12.1)
Non-ethnic minorities	108 (24.0)	153 (34.0)	111 (24.7)	44 (9.8)	34 (7.6)
Test	*X*^2^ = 8.905, *p* = 0.064				
**Different regions**					
Eastern	4 (7.5)	11 (20.8)	26 (49.1)	3 (5.7)	9 (17.0)
Central	18 (40.0)	17 (37.8)	4 (8.9)	2 (4.4)	4 (8.9)
Western	112 (25.3)	144 (32.5)	102 (23.0)	53 (12.0)	32 (7.2)
Test	*X*^2^ = 40.199, *p* < 0.001^***^				
**Different levels of economic development**					
Underdeveloped	97 (32.7)	97 (32.7)	58 (19.5)	24 (8.1)	21 (7.1)
General	23 (14.5)	54 (34.0)	49 (30.8)	20 (12.6)	13 (8.2)
Relatively developed villages	14 (16.5)	21 (24.7)	25 (29.4)	14 (16.5)	11 (12.9)
Test	*X*^2^ = 48.554, *p* < 0.001^***^				
Aggregation	134 (24.8)	172 (31.8)	132 (24.4)	58 (10.7)	45 (8.3)

^*^p < 0.05, ^**^p < 0.01, ^***^p < 0.001.

#### 4.2.5 Analysis of differences in the matching degree of the organization of sports activities for older adults

As shown in Table 5:

(1) The overall assessment of the matching degree of the organization of sports activities for older adults is that 28.7% of the respondents think it is “basic match,” 10.5% think it is “comparative match,” and 10.5% think it is “complete match.” The value of “matching baseline” is 49.7%, failing to meet the standard.
(2) There was no significant difference in the matching degree of the organization of sports activities for older adults between villages of different ethnic groups (*X*^2^ = 8.396, *p* = 0.078 > 0.05), but there were significant differences between different regions and levels of economic development (*X*^2^ = 25.914, *p* = 0.001 < 0.01; *X*^2^ = 48.554, *p* = 0.000 < 0.01). Specifically, the value of “matching baseline” for villages in the eastern region (75.5%) was significantly higher than that in the western region (48.7%), while that in the western region was significantly higher than that in the central region (28.8%). The value of “matching baseline” in the “relatively developed” villages (68.2%) was significantly higher than that in the “general” villages (57.9%), while that in the “general” villages was significantly higher than that in the “underdeveloped” villages (40.1%).

**Table 5:** Analysis of difference in the matching degree of the organization of sports activities for older adults among different villages.

**(*N* = 541)**	**Complete mismatch**	**Mismatch**	**Basic match**	**Comparative match**	**Complete match**
**Different ethnic groups**					
Ethnic minorities	28 (30.8)	16 (17.6)	22 (24.2)	12 (13.2)	13 (14.3)
Non-ethnic minorities	101 (22.4)	127 (28.2)	133 (29.6)	45 (10.0)	44 (9.8)
Test	*X*^2^ = 8.396, *p* = 0.078				
**Different regions**					
Eastern	5 (9.4)	8 (15.0)	25 (47.2)	7 (13.2)	8 (15.1)
Central	18 (40.0)	14 (31.1)	6 (13.3)	2 (4.4)	5 (11.1)
Western	106 (23.9)	121 (27.3)	124 (28.0)	48 (10.8)	44 (9.9)
Test	*X*^2^ = 25.914, *p* < 0.001^***^				
**Different levels of economic development**					
Underdeveloped	92 (31.0)	86 (29.0)	66 (22.2)	27 (9.1)	26 (8.8)
General	22 (13.8)	45 (28.3)	54 (34.0)	18 (11.3)	20 (12.6)
Relatively developed villages	15 (17.65)	12 (14.1)	35 (41.2)	12 (14.1)	11 (12.9)
Test	*X*^2^ = 48.554, *p* < 0.001^***^				
Aggregation	129 (23.8)	143 (26.4)	155 (28.7)	57 (10.5)	57 (10.5)

^*^p < 0.05, ^**^p < 0.01, ^***^p < 0.001.

#### 4.2.6 Analysis of differences in the matching degree of the guidance for older adults participating in sports activities

As shown in Table 6:

(1) The overall assessment of the matching degree of the guidance for older adults participating in sports activities is that 25.7% of the respondents think it is “basic match,” 13.3% think it is “comparative match,” and 9.1% think it is “complete match.” The value of “matching baseline” is 48.1%, failing to meet the standard.
(2) There was no significant difference in the matching degree of the guidance for older adults participating in sports activities between villages of different ethnic groups (*X*^2^ = 8.515, *p* = 0.074 > 0.05), but there were significant differences between different regions and levels of economic development (*X*^2^ = 34.904, *p* = 0.000 < 0.01; *X*^2^ = 41.043, *p* = 0.000 < 0.01). Specifically, the value of “matching baseline” for villages in the eastern region (77.4%) was significantly higher than that in the western region (46.5%), while that in the western region was significantly higher than that in the central region (28.8%). The value of “matching baseline” in the “relatively developed” villages (66.8%) was significantly higher than that in the “general” villages (55.4%), while that in the “general” villages was significantly higher than that in the “underdeveloped” villages (38.7%).

**Table 6:** Analysis of difference in the matching degree of the guidance for older adults participating in sports activities among different villages.

**(*N* = 541)**	**Complete mismatch**	**Mismatch**	**Basic match**	**Comparative match**	**Complete match**
**Different ethnic groups**					
Ethnic minorities	24 (26.4)	20 (22.0)	18 (19.8)	17 (18.7)	12 (13.2)
Non-ethnic minorities	99 (22.0)	138 (30.7)	121 (26.9)	55 (12.2)	37 (8.2)
Test	*X*^2^ = 8.515, *p* = 0.074				
**Different regions**					
Eastern	4 (7.5)	8 (15.1)	26 (49.1)	8 (15.1)	7 (13.2)
Central	18 (40.0)	14 (31.1)	7 (15.6)	1 (2.2)	5 (11.1)
Western	101 (22.8)	136 (30.7)	106 (23.9)	63 (14.2)	37 (8.4)
Test	*X*^2^ = 34.904, *p* < 0.001^***^				
**Different levels of economic development**					
Underdeveloped	91 (30.6)	91 (30.6)	54 (18.2)	36 (12.1)	25 (8.4)
General	19 (11.9)	52 (32.7)	52 (32.7)	20 (12.6)	16 (10.1)
Relatively developed villages	13 (15.3)	15 (17.6)	33 (38.8)	16 (18.6)	8 (9.4)
Test	*X*^2^ = 41.043, *p* < 0.001^***^				
**Aggregate**	123 (22.7)	158 (29.2)	139 (25.7)	72 (13.3)	49 (9.1)

^*^p < 0.05, ^**^p < 0.01, ^***^p < 0.001.

#### 4.2.7 Analysis of differences in the matching degree of the provision of health and fitness knowledge for older adults

As shown in Table 7:

(1) The overall assessment of the matching degree of the provision of health and fitness knowledge for older adults is that 27.4% of the respondents think it is “basic match,” 15.2% think it is “comparative match,” and 13.3% think it is “complete match.” The value of “matching baseline” is 55.9%, failing to meet the standard.
(2) There was no significant difference in the matching degree of the provision of health and fitness knowledge for older adults between villages of different ethnic groups (*X*^2^ = 9.171, *p* = 0.057 > 0.05), but there were significant differences between different regions and levels of economic development (*X*^2^ = 40.037, *p* = 0.000 < 0.01; *X*^2^ = 39.307, *p* = 0.000 < 0.01). Specifically, the value of “matching baseline” for villages in the eastern region (77.4%) was significantly higher than that in the western region (55.6%), while that in the western region was significantly higher than that in the central region (33.4%). There was no significant difference between the “matching baseline” values of “relatively developed” and “general” villages (70.6 vs. 66.6%), but both were significantly higher than those of “underdeveloped” villages (45.8%).

**Table 7:** Analysis of difference in the matching degree of the provision of health and fitness knowledge for older adults among different villages.

**(*N* = 541)**	**Complete mismatch**	**Mismatch**	**Basic match**	**Comparative match**	**Complete match**
**Different ethnic groups**					
Ethnic minorities	23 (25.3)	19 (20.9)	20 (22.0)	10 (11.0)	19 (20.9)
Non-ethnic minorities	85 (18.9)	112 (24.9)	128 (28.4)	72 (16.0)	53 (11.8)
Test	*X*^2^ = 9.171, *p* = 0.057				
**Different regions**					
Eastern	4 (7.5)	8 (15.1)	30 (56.6)	3 (5.7)	8 (15.1)
Central	17 (37.8)	13 (28.9)	7 (15.6)	3 (6.7)	5 (11.1)
Western	87 (19.6)	110 (24.8)	111 (25.1)	76 (17.2)	59 (13.3)
Test	*X*^2^ = 40.037, *p* < 0.001^***^				
**Different levels of economic development**					
Underdeveloped	80 (26.9)	81 (27.3)	63 (21.2)	37 (12.5)	36 (12.1)
General	19 (11.9)	34 (21.4)	59 (37.1)	22 (13.8)	25 (15.7)
Relatively developed villages	9 (10.6)	16 (18.8)	26 (30.6)	23 (27.1)	11 (12.9)
Test	*X*^2^ = 39.307, *p* < 0.001^***^				
Aggregation	108 (20.0)	131 (24.2)	148 (27.4)	82 (15.2)	72 (13.3)

^*^p < 0.05, ^**^p < 0.01, ^***^p < 0.001.

#### 4.2.8 Analysis of differences in the matching degree of the publicity and mobilization for older adults to participate in sports

As shown in Table 8:

(1) The overall assessment of the matching degree of the publicity and mobilization for older adults to participate in sports is that 30.1% of the respondents think it is “basic match,” 13.9% think it is “comparative match,” and 10.7% think it is “complete match.” The value of “matching baseline” is 54.7%, failing to meet the standard.
(2) There was no significant difference in the matching degree of the publicity and mobilization for older adults to participate in sports between villages of different ethnic groups (*X*^2^ = 6.995, *p* = 0.136 > 0.05), but there were significant differences between different regions and levels of economic development (*X*^2^ = 28.87, *p* = 0.000 < 0.01; *X*^2^ = 54.324, *p* = 0.000 < 0.01). Specifically, the value of “matching baseline” for villages in the eastern region (71.7%) was significantly higher than that in the western region (54.9%), while that in the western region was significantly higher than that in the central region (33.3%). The value of “matching baseline” in the “relatively developed” villages (74.2%) was significantly higher than that in the “general” villages (66.0%), while that in the “general” villages was significantly higher than that in the “underdeveloped” villages (43.1%).

**Table 8:** Analysis of difference in the matching degree of the publicity and mobilization for older adults among different villages.

**(*N* = 541)**	**Complete mismatch**	**Mismatch**	**Basic match**	**Comparative match**	**Complete match**
**Different ethnic groups**					
Ethnic minorities	21 (23.1)	24 (26.4)	19 (20.9)	12 (13.2)	15 (16.5)
Non-ethnic minorities	90 (20.0)	110 (24.4)	144 (32.0)	63 (14.0)	43 (9.6)
Test	*X*^2^ = 6.995, *p* = 0.136				
**Different regions**					
Eastern	3 (5.7)	12 (22.6)	25 (47.2)	6 (11.3)	7 (13.2)
Central	19 (42.2)	11 (24.4)	7 (15.6)	2 (4.4)	6 (13.3)
Western	89 (20.1)	111 (25.1)	131 (29.6)	67 (15.1)	45 (10.2)
Test	*X*^2^ = 28.87, *p* < 0.001^***^				
**Different levels of economic development**					
Underdeveloped	86 (29.0)	83 (27.9)	62 (20.9)	32 (10.8)	34 (11.4)
General	15 (9.4)	39 (24.5)	67 (42.1)	24 (15.1)	14 (8.8)
Relatively developed villages	10 (11.8)	12 (14.1)	34 (40.0)	19 (22.4)	10 (11.8)
Test	*X*^2^ = 54.324, *p* < 0.001^***^				
Aggregation	111 (20.5)	134 (24.8)	163 (30.1)	75 (13.9)	58(10.7)

^*^p < 0.05, ^**^p < 0.01, ^***^p < 0.001.

#### 4.2.9 Analysis of differences in the matching degree of the system construction of sports for older adults

As shown in Table 9:

(1) The overall assessment of the matching degree of the system construction of sports for older adults is that 34.1% of the respondents think it is “basic match,” 18.8% think it is “comparative match,” and 9.4% think it is “complete match.” The value of “matching baseline” is 62.3%, which basically meets the standard.
(2) There was no significant difference in the matching degree of the system construction of sports for older adults between villages of different ethnic groups (*X*^2^ = 7.31, *p* = 0.081 > 0.05), but there were significant differences between different regions and levels of economic development (*X*^2^ = 49.118, *p* = 0.000 < 0.01; *X*^2^ = 48.996, *p* = 0.000 < 0.01). Specifically, the value of “matching baseline” for villages in the eastern region (68.0%) was significantly higher than that in the western region (44.0%), while that in the western region was significantly higher than that in the central region (24.4%). The value of “matching baseline” in the “relatively developed” villages (62.3%) was significantly higher than that in the “general” villages (53.4%), while that in the “general” villages was significantly higher than that in the “underdeveloped” villages (35.0%).

**Table 9:** Analysis of difference in the matching degree of the system construction of sports for older adults among different villages.

**(*N* = 541)**	**Complete mismatch**	**Mismatch**	**Basic match**	**Comparative match**	**Complete match**
**Different ethnic groups**					
Ethnic minorities	23 (25.3)	26 (28.6)	19 (20.9)	7 (7.7)	16 (17.6)
Non-ethnic minorities	103 (22.9)	147 (32.7)	108 (24.0)	58 (12.9)	34 (7.6)
Test	*X*^2^ = 7.31, *p* = 0.081				
**Different regions**					
Eastern	4 (7.5)	13 (24.5)	25 (47.2)	1 (1.9)	10 (18.9)
Central	20 (44.4)	14 (31.1)	3 (6.7)	2 (4.4)	6 (13.3)
Western	102 (23.0)	146 (33.0)	99 (22.3)	62 (14.0)	34 (7.7)
Test	*X*^2^ = 49.118, *p* < 0.001^***^				
**Different levels of economic development**					
Underdeveloped	96 (32.3)	97 (32.7)	46 (15.5)	30 (10.1)	28 (9.4)
General	17 (10.7)	57 (35.8)	52 (32.7)	19 (11.9)	14 (8.8)
Relatively developed villages	13 (15.3)	19 (22.4)	29 (34.1)	16 (18.8)	8 (9.4)
Test	*X*^2^ = 48.996, *p* < 0.001^***^				
Aggregation	126 (23.3)	173 (32.0)	127 (23.5)	65 (12.0)	50 (9.2)

^*^p < 0.05, ^**^p < 0.01, ^***^p < 0.001.

#### 4.2.10 Analysis of differences in the matching degree of the physical fitness monitoring service for older adults

As shown in Table 10:

(1) The overall assessment of the matching degree of the physical fitness monitoring service for older adults is that 28.2% of the respondents think it is “basic match,” 16.5% think it is “comparative match,” and 14.6% think it is “complete match.” The value of “matching baseline” is 59.3%, which is close to the standard.
(2) There was no significant difference in the matching degree of the system construction of sports for older adults between villages of different ethnic groups (*X*^2^ = 3.199, *p* = 0.525 > 0.05), but there were significant differences between different regions and levels of economic development (*X*^2^ = 35.696, *p* = 0.000 < 0.01; *X*^2^ = 18.743, *p* = 0.016 < 0.05). Specifically, the value of “matching baseline” for villages in the eastern region (77.4%) was significantly higher than that in the western region (56.9%), while that in the western region was significantly higher than that in the central region (35.6%). The value of “matching baseline” in the “relatively developed” villages (68.2%) was significantly higher than that in the “general” villages (61.7%), while that in the “general” villages was significantly higher than that in the “underdeveloped” villages (51.5%).

**Table 10:** Analysis of difference in the matching degree of the physical fitness monitoring service for older adults among different villages.

**(*N* = 541)**	**Complete mismatch**	**Mismatch**	**Basic match**	**Comparative match**	**Complete match**
**Different ethnic groups**					
Ethnic minorities	19 (20.9)	18 (19.8)	23 (25.3)	13 (14.3)	18 (19.8)
Non-ethnic minorities	85 (18.9)	110 (24.4)	118 (26.2)	76 (16.9)	61 (13.6)
Test	*X*^2^ = 3.199, *p* = 0.525				
**Different regions**					
Eastern	4 (7.5)	8 (15.1)	26 (49.1)	7 (13.2)	8 (15.1)
Central	19 (42.2)	10 (22.2)	4 (8.9)	5 (11.1)	7 (15.6)
Western	81 (18.3)	110 (24.8)	111 (25.1)	77 (17.4)	64 (14.4)
Test	*X*^2^ = 35.696, *p* < 0.001^***^				
**Different levels of economic development**					
Underdeveloped	73 (24.6)	71 (23.9)	65 (21.9)	46 (15.5)	42 (14.1)
General	21 (13.2)	40 (25.2)	52 (32.7)	23 (14.5)	23 (14.5)
Relatively developed villages	10 (11.8)	17 (20.0)	24 (28.2)	20 (23.5)	14 (16.5)
Test	*X*^2^ = 18.743, *p* < 0.001^***^				
**Aggregate**	104 (19.2)	128 (23.7)	141 (26.1)	89 (16.5)	79 (14.6)

^*^p < 0.05, ^**^p < 0.01, ^***^p < 0.001.

### 4.3 Correlation analysis between the matching of supply and demand, and its related factors of public sports services for rural older adults

To construct a database suitable for CCA, the two independent samples (demand-side survey of older adults and supply-side survey of village leaders) were merged. To address the different sample sizes (*N* = 1,667 for older adults vs. *N* = 563 for village leaders), a stratified random sampling approach was used to create a matched dataset. Following the regional distribution of the village leader sample, we randomly selected 32.7%, 34.5%, and 33.9% of the older adult samples from the eastern, central, and western regions, respectively, to balance the sample sizes for the analysis. To validate this method and ensure that the random sampling did not introduce bias, we performed Chi-square tests comparing the demographic distribution (e.g., gender, age group, and income level) of the selected sub-sample with the original full sample of older adults. The results showed no statistically significant differences, confirming that the sub-sample retained the characteristics of the original population and was suitable for the subsequent integrated analysis.

#### 4.3.1 Correlation between the health status, lifestyle of rural older adults and the matching of supply and demand of public sports services

**Figure 2:**
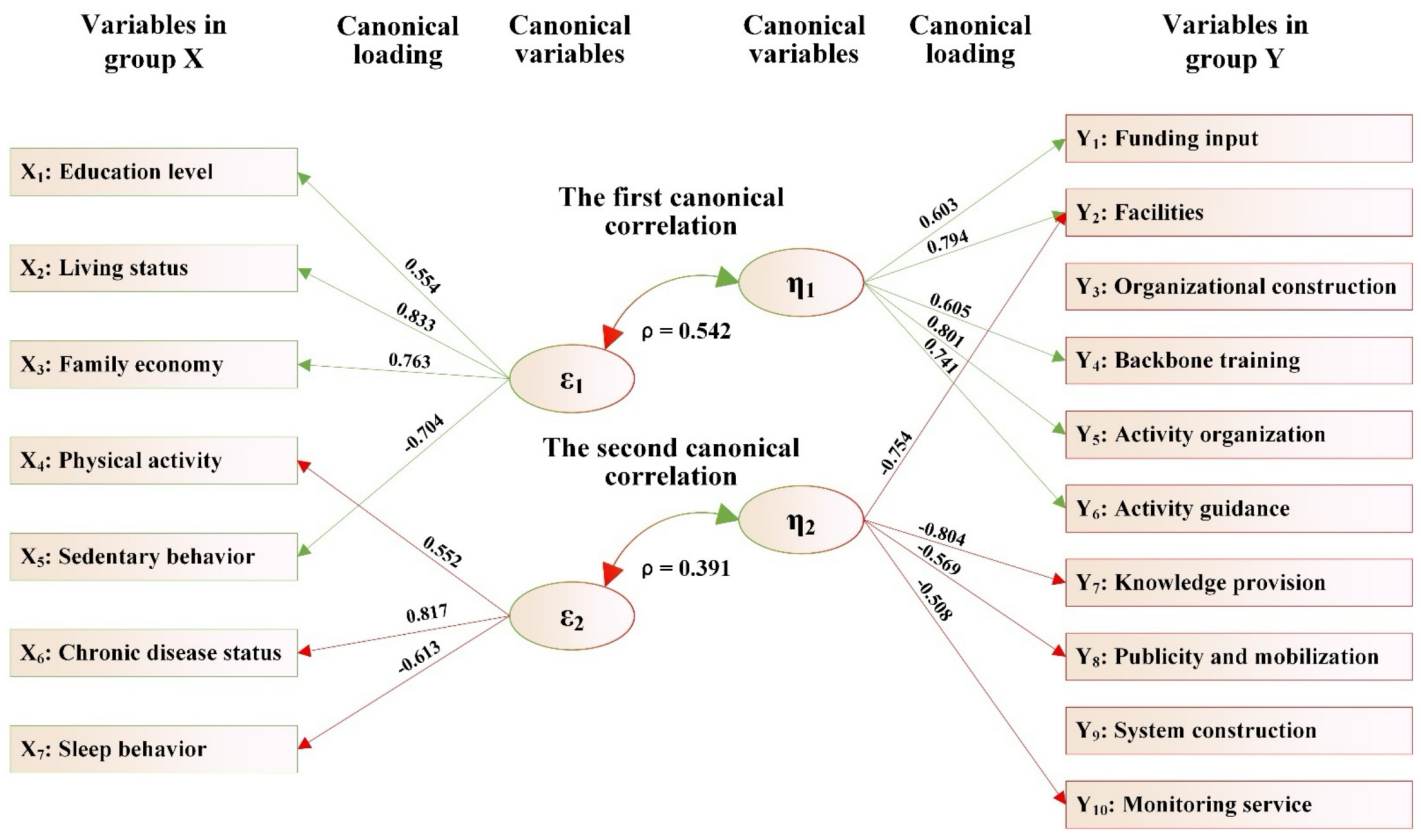
The canonical correlation path between the health status, lifestyle of rural older adults and the matching of supply and demand of public sports services.

As shown in Figure 2 and Table 11:

(1) The variables in group X consist of seven variables X_1_-X_7_, which can be extracted into two canonical variables (ε_1_ and ε_2_). The variables in group Y consist of 10 items related to the matching of supply and demand for public sports services (Y_1_-Y_10_) and also extract two canonical variables (η_1_ and η_2_). Two groups of canonical variables form two pairs of canonical correlations, namely the first canonical correlation (ε_1_ and η_1_) and the second canonical correlation (ε_2_ and η_2_). Both canonical correlation coefficients (ρ) reached the significant level, namely ρ_1_ = 0.542 (*p* < 0.001) and ρ_2_ = 0.391 (*p* < 0.001). The amount that can be explained (ρ_2_) was 29.38 and 15.29%, respectively, which means that the two groups of canonical variables ε_1_ and η_1_, ε_2_ and η_2_ can mutually explain 29.38 and 15.29% of each other's total variance, respectively.
(2) In the first canonical correlation, the canonical variable ε_1_ derived from group X can explain 29.38% of the total variance of the canonical variable η_1_ in group Y. Meanwhile, the first canonical variable (η_1_) derived from group Y can explain 57.26% of the total variance of the variables in group Y. Therefore, the variables in group X, through their first canonical variable (ε_1_), can explain 16.82% of the total variance of the variables in group Y (i.e., overlapping variance = 29.38% × 57.26% = 16.82%). Similarly, according to this calculation method, the variables in group Y, *via* their first canonical variable (η_1_), can explain 12.27% of the total variance of the variables in group X.

**Table 11:** The canonical correlation analysis between the health status, lifestyle of rural older adults and the matching of supply and demand of public sports services.

**Variables**	**Canonical variables**		**Variables**	**Canonical variables**	
**(X)**	ε_1_	ε_2_	**(Y)**	η_1_	η_2_
X_1_: Education level	0.554	0.055	Y_1_: Funding input	0.603	0.127
X_2_: Living status	0.833	0.033	Y_2_: Facilities	0.794	−0.754
X_3_: Family economy	0.763	0.163	Y_3_: Organizational construction	0.087	0.164
X_4_: Physical activity	0.152	0.552	Y_4_: Backbone training	0.605	0.088
X_5_: Sedentary behavior	−0.704	−0.147	Y_5_: Activity organization	0.801	0.341
X_6_: Chronic disease status	−0.161	0.817	Y_6_: Activity guidance	0.741	0.058
X_7_: Sleep behavior	0.045	−0.613	Y_7_: Knowledge provision	0.065	−0.804
			Y_8_: Publicity and mobilization	0.119	−0.569
			Y_9_: System construction	0.103	0.091
			Y_10_: Monitoring service	0.154	−0.508
Sampling variation (%)	41.75	27.74		57.26	31.44
Overlapping Variance (%)	12.27	4.24		16.82	4.81
Canonical correlation coefficient (ρ)	0.542^***^	0.391^***^			
Mutual explained variance (ρ^2^)	0.2938	0.1529			
Significance level test	0.000	0.000			

^*^p < 0.05, ^**^p < 0.01, ^***^p < 0.001.

In this canonical correlation, among the variables in group X, four variables, including “educational level,” “residential status,” “family economic status,” and “sedentary behavior,” have absolute values of coefficients of the first canonical variable (ε_1_) that are all ≥0.30, which are 0.554, 0.833, 0.763, and −0.704, respectively. In the variables in group Y, five variables, including “funding input,” “facilities,” “backbone training,” “activity organization,” and “activity guidance,” have absolute values of coefficients in the first canonical variable (η_1_) that are all ≥0.30, which are 0.603, 0.794, 0.605, 0.801, and 0.741, respectively. This indicates that the four variables (“educational level,” “living status,” “family economy,” and “sedentary behavior”) of older adults can establish a significant correlation with the five variables (“funding input,” “facilities,” “backbone training,” “activity organization,” and “activity guidance”) reflecting the equilibrium degree of supply-demand matching in public sports services for rural older adults through the first canonical correlation (ε_1_ and η_1_).

(3) In the second canonical correlation, the canonical variable ε_2_ derived from the variables in group X can explain 15.29% of the total variance of the canonical variable η_2_ in the variables in group Y. Meanwhile, the second canonical variable (η_2_) of the variables in group Y can account for 31.44% of the total variance of the variables in group Y. Therefore, the variables in group X, through their second canonical variable (ε_2_), can explain 4.81% of the total variance of the variables in group Y. Similarly, according to this calculation method, the variables in group Y, *via* their second canonical variable (η_2_), can explain 4.24% of the total variance of the variables in group X.

In this canonical correlation, among the variables in group X, three variables, including “physical activity,” “chronic disease status,” and “sleep behavior,” have absolute values of the coefficients of the second canonical variable (ε_2_) that are ≥0.30, which are 0.552, 0.817, and −0.613, respectively. In the variables in group Y, four variables, including “facilities,” “knowledge provision,” “publicity and mobilization,” and “monitoring service,” have absolute values of coefficients in the second canonical variable (η_2_) that are all ≥0.30, which are −0.754, −0.804, −0.569, and −0.508, respectively. This indicates that the three variables (“physical activity,” “chronic disease status,” and “sleep behavior”) of older adults can establish a significant correlation with the four variables (“facilities,” “knowledge provision,” “publicity and mobilization,” and “monitoring service”) reflecting the equilibrium degree of supply-demand matching in public sports services for rural older adults through the second canonical correlation (ε_2_ and η_2_).

(4) From the absolute value and positive/negative sign of the variable coefficients contributing to the first canonical correlation (ε_1_ and η_1_), it is easy to observe that in villages where the equilibrium degree of supply-demand matching for public sports services for rural older adults, such as “funding input,” “facilities,” “backbone training,” “activity organization,” and “activity guidance” is relatively high, the older adults have a higher “educational level,” better “living status,” and more affluent “family economy.” However, older adults in these villages suffer from a serious problem of “sedentary behavior.”

From the absolute values and positive/negative signs of the variable coefficients contributing to the second canonical correlation (ε_2_ and η_2_), the equilibrium degree of matching for public sports services in terms of “facilities,” “knowledge provision,” “publicity and mobilization,” and “monitoring service” shows an inverse relationship with older adults' “physical activities” and “chronic disease status,” while showing a positive relationship with their “sleep behaviors.” This finding is quite strange. This counterintuitive finding does not imply that poor services improve health. Instead, it likely reflects the complex reality of rural life: in villages with scarce public sports facilities and services, older adults' primary form of “physical activity” often consists of strenuous, non-leisure agricultural or domestic labor. This type of physically demanding lifestyle is associated with a high incidence of chronic diseases, poor sleep quality, and a lack of formal sports services, thus explaining the negative statistical correlation. This highlights a critical misallocation: service supply is lowest where health needs are greatest.

In addition, the percentage of overlapping variance showed that the overlapping variances under the two canonical variables ε_1_ and ε_2_ in group X were 12.27 and 4.24% (sum 16.51%), and the overlapping variances under the two canonical variables η_1_ and η_2_ in group Y were 16.82 and 4.81% (sum 21.63%), which indicates that the canonical variables ε_1_ and ε_2_ of group X can jointly explain 21.63% of the variance of the 10 variables in group Y, while η_1_ and η_2_ in group Y can only jointly account for 16.51% of the variance of the seven variables in group X. Evidently, from these two groups of correlation relationships, it is not difficult to observe that the variables in group X essentially pertain to the issue of the demands of older adults. The overlapping variance indicates that the current match of supply and demand of public sports services for rural older adults cannot yet meet the needs of older adults in terms of health status and lifestyle, and it also highlights the inadequacy in the supply-demand matching degree of public sports services for rural older adults.

#### 4.3.2 Correlation between rural older adults' awareness of physical activity and the matching of supply and demand of public sports services

**Figure 3:**
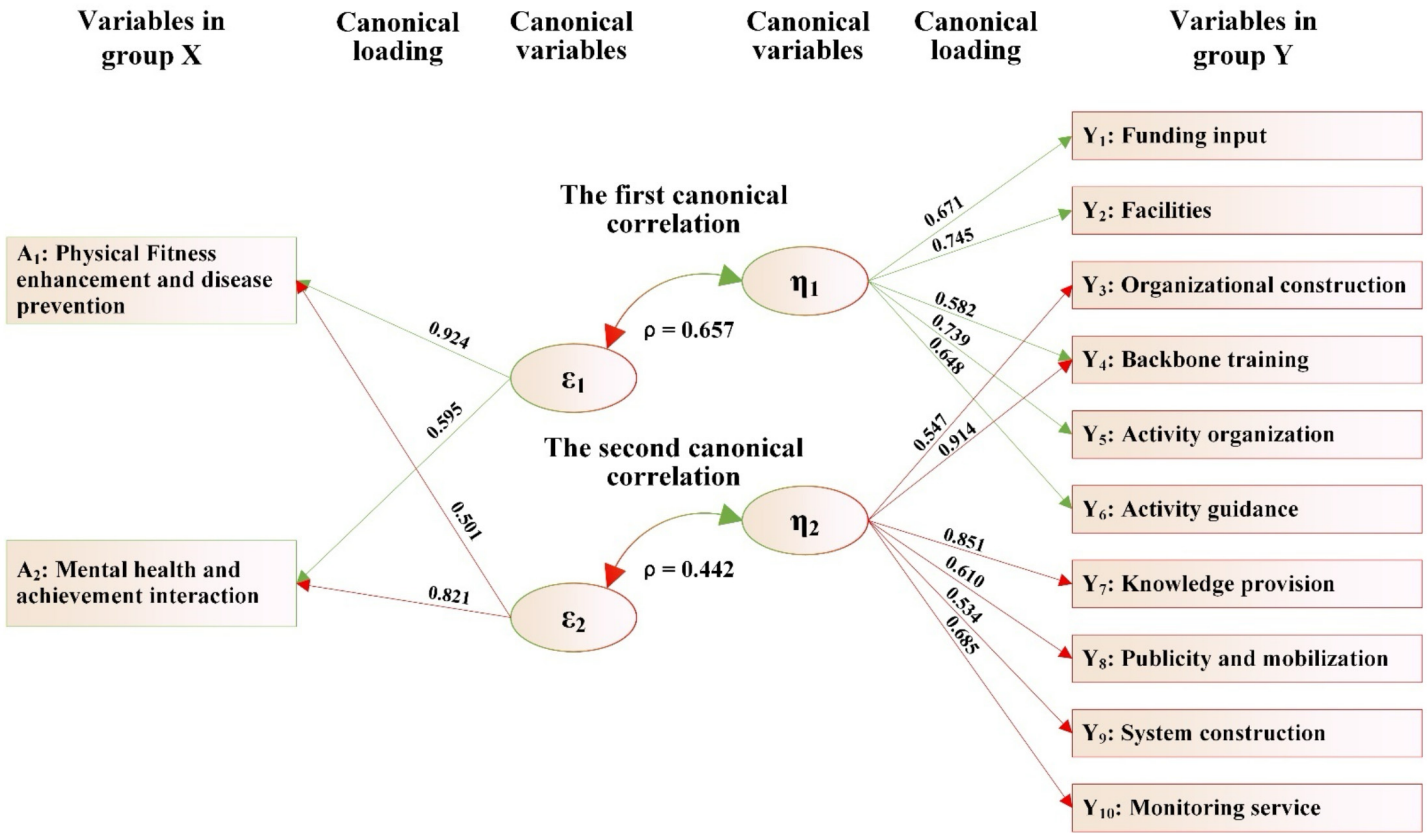
The canonical correlation path between rural older adults' awareness of physical activity and the matching of supply and demand of public sports services.

As Figure 3 shows, the combined Table 12 can be found:

(1) The two canonical variables (ε_1_ and ε_2_) extracted from the variables in group X (including two dimensions: A_1_ and A_2_) and the two canonical variables (η_1_ and η_2_) extracted from the variables in group Y (Y_1_-Y_10_) form the first canonical correlation (ε_1_ and η_1_) and the second canonical correlation (ε_2_ and η_2_), respectively.
(2) In the first canonical correlation, the canonical correlation coefficient ρ = 0.657^***^, indicating that the canonical variable (ε_1_) derived from group X can explain 43.16% of the total variance of the canonical variable (η_1_) in group Y. Meanwhile, the first canonical variable (η_1_) of the variables in group Y can account for 57.26% of the total variance of the variables in group Y. Therefore, the variables in group X, through their first canonical variable (ε_1_), can explain 23.83% of the total variance of the variables in group Y. Similarly, according to this calculation method, the variables in group Y, *via* their first canonical variable (η_1_), can explain 22.15% of the total variance of the variables in group X.

**Table 12:** The canonical correlation analysis table between the awareness of physical exercise of rural older adults and the matching between supply and demand of public sports services.

**Variables**	**Canonical variables**		**Variables**	**Canonical variables**	
**(X)**	ε_1_	ε_2_	**(Y)**	η_1_	η_2_
A_1_: Physical Fitness enhancement and disease prevention	0.924	0.501	Y_1_: Funding input	0.671	0.218
A_2_: Mental health and achievement interaction	0.595	0.821	Y_2_: Facilities	0.745	0.157
			Y_3_: Organizational construction	0.223	0.547
			Y_4_: Backbone training	0.582	0.914
			Y_5_: Activity organization	0.739	0.301
			Y_6_: Activity guidance	0.648	0.188
			Y_7_: Knowledge provision	0.023	0.851
			Y_8_: Publicity and mobilization	0.340	0.610
			Y_9_: System construction	0.188	0.534
			Y_10_: Monitoring service	0.046	0.685
Sampling variation (%)	51.33	33.24		57.21	37.06
Overlapping Variance (%)	22.15	6.50		23.83	7.24
Canonical correlation coefficient (ρ)	0.657^***^	0.442^***^			
Mutual explained variance (ρ^2^)	0.4316	0.1954			
Significance level test	0.000	0.000			

^*^p < 0.05, ^**^p < 0.01, ^***^p < 0.001.

In this canonical correlation, among the variables in group X, two variables, including “physical fitness enhancement and disease prevention” and “mental health and achievement interaction,” have absolute values of coefficients of the first canonical variable (ε_1_) that are all ≥0.30, which are 0.924 and 0.595, respectively. In the variables in group Y, five variables, including “funding input,” “facilities,” “organizational construction,” “activity organization,” and “activity guidance,” have absolute values of coefficients in the first canonical variable (η_1_) that are all ≥0.30, which are 0.671, 0.745, 0.582, 0.739, and 0.648, respectively. It shows that to improve older adults' awareness that physical exercise is beneficial to enhance physical fitness and prevent diseases as well as mental health and achievement interaction, a significant positive correlation can be generated with the equilibrium degree of supply-demand matching in public sports services such as “funding input,” “facilities,” “organizational construction,” “activity organization,” and “activity guidance” through the first canonical correlation (ε_1_ and η_1_).

(3) In the second canonical correlation, the canonical variable (ε_2_) derived from the variables in group X can explain 19.54% of the total variance of the canonical variable (η_2_) in the variables in group Y. Meanwhile, the second canonical variable (η_2_) of the variables in group Y can account for 37.06% of the total variance of the variables in group Y. Therefore, the variables in group X, through their second canonical variable (ε_2_), can explain 7.24% of the total variance of the variables in group Y. Similarly, according to this calculation method, the variables in group Y, *via* their second canonical variable (η_2_), can explain 6.5% of the total variance of the variables in group X.

In this canonical correlation, among the variables in group X, two variables, including “physical fitness enhancement and disease prevention” and “mental health and achievement interaction,” have absolute values of the coefficients of the second canonical variable (ε_2_) that are ≥0.30, which are 0.501 and 0.821, respectively. In the variables in group Y, six variables, including “organizational construction,” “backbone training,” “knowledge provision,” “publicity and mobilization,” and “monitoring service,” have absolute values of coefficients in the second canonical variable (η_2_) that are all ≥0.30, which are 0.914, 0.851, 0.610, 0.534, and 0.685, respectively. It shows that to improve older adults' awareness that physical exercise is beneficial to enhance physical fitness and prevent diseases as well as mental health and achievement interaction, a significant positive correlation can be generated with the equilibrium degree of supply-demand matching in public sports services such as “organizational construction,” “backbone training,” “knowledge provision,” “publicity and mobilization,” and “monitoring service” through the second canonical correlation (ε_2_ and η_2_).

(4) From the first canonical correlation (ε_1_ and η_1_, ρ = 0.657, *p* < 0.001), the variable that contributes significantly to ε_1_ is “physical fitness enhancement and disease prevention” (0.924), while the variables that contribute significantly to η_1_ are “facilities” (0.745) and “activity organization” (0.739). The standardized coefficients of the variables contributing to both ε_1_ and η_1_ are also in the same direction. This indicates that a higher degree of supply-demand matching for tangible services like sports facilities and organized events is significantly associated with a stronger belief among older adults that exercise can improve their health. The high loadings of these two factors in particular suggest their critical role in shaping health cognition. This empirical finding directly informs our policy recommendation to prioritize investment in facility construction and the regular organization of activities, as these appear to be the most effective channels for improving health awareness.

From the second canonical correlation (ε_2_ and η_2_, ρ = 0.442, *p* < 0.001), the variant that makes an important contribution to ε_2_ is “mental health and achievement interaction” (0.821), and the variant that makes an important contribution to η_2_ are “backbone training” (0.914) and “knowledge provision” (0.851). The standardized coefficients of the variables contributing to both ε_2_ and η_2_ are also in the same direction. This indicates that the higher the supply-demand matching degree in many aspects, such as “training of key members” and “knowledge provision” in public sports services for older adults, the more beneficial it is to improve older adults' cognitive ability regarding the role of physical exercise in “mental health and achievement interaction.”

In addition, the percentage of overlapping variance shows that the canonical variables ε_1_ and ε_2_ in group X can explain 31.07% (23.83% + 7.24%) of the 10 variables in group Y, while the canonical variables η_1_ and η_2_ in group Y can explain 28.65% (22.15% + 6.50%) of the two variables in group X. Obviously, the difference between the two percentages of overlapping variance is not significant, which indicates that the current supply-demand matching of rural public sports services has basically met the needs of older adults in terms of their cognition of physical exercise.

#### 4.3.3 Correlation between impediments to physical activity of rural older adults and the matching of supply and demand of public sports services

**Figure 4:**
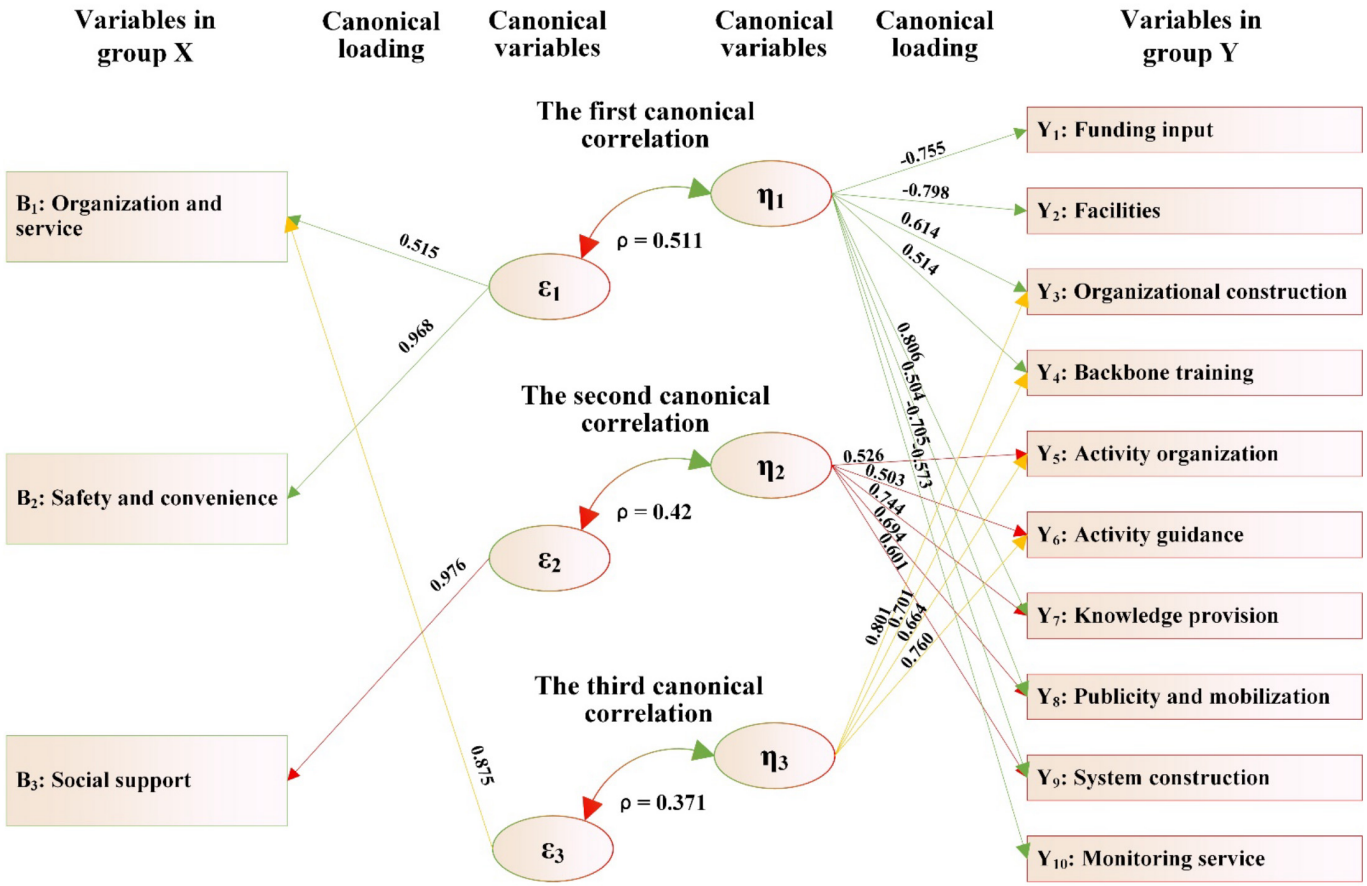
The canonical correlation path between impediments to physical activity of rural older adults and the matching of supply and demand of public sports services.

Figure 4, given in Table 13, can be found:

(1) The three canonical variables (ε_1_, ε_2_, and ε_3_) extracted from the variables in group X (including three dimensions: B1, B2, and B3) and the two canonical variables (η_1_, η_2_, and η_3_) extracted from the variables in group Y (Y_1_-Y_10_) form the first canonical correlation (ε_1_ and η_1_), the second canonical correlation (ε_2_ and η_2_), and the third canonical correlation (ε_3_ and η_3_).
(2) In the first canonical correlation, the canonical correlation coefficient ρ = 0.511^***^, indicating that the canonical variable (ε_1_) derived from group X can explain 26.11% of the total variance of the canonical variable (η_1_) in group Y. Meanwhile, the first canonical variable (η_1_) of the variables in group Y can account for 45.15% of the total variance of the variables in group Y. Therefore, the variables in group X, through their first canonical variable (ε_1_), can explain 11.79% of the total variance of the variables in group Y. Similarly, according to this calculation method, the variables in group Y, *via* their first canonical variable (η_1_), can explain 10.48% of the total variance of the variables in group X.

**Table 13:** The canonical correlation analysis table between obstructive factors of physical exercise of rural older adults and the matching between supply and demand of public sports services.

**Variables**	**Canonical variables**			**Variables**	**Canonical variables**		
**(X)**	ε_1_	ε_2_	ε_3_	**(Y)**	η_1_	η_2_	η_3_
B_1_: Organization and service	0.515	0.234	0.875	Y_1_: Funding input	−0.755	−0.339	−0.165
B_2_: Safety and convenience	0.968	0.118	0.133	Y_2_: Facilities	−0.798	−0.129	0.108
B_3_: Social support	0.192	0.976	0.100	Y_3_: Organizational construction	0.614	0.183	0.801
				Y_4_: Backbone training	0.514	0.092	0.664
				Y_5_: Activity organization	0.067	0.526	0.701
				Y_6_: Activity guidance	0.159	0.503	0.760
				Y_7_: Knowledge provision	0.806	0.744	−0.102
				Y_8_: Publicity and mobilization	0.504	0.694	0.148
				Y_9_: System construction	−0.705	0.601	0.206
				Y_10_: Monitoring service	−0.573	0.126	−0.178
Sampling variation (%)	40.14	28.69	17.54		45.15	30.77	21.65
Overlapping Variance (%)	10.48	5.06	2.41		11.79	5.43	2.98
Canonical correlation coefficient (ρ)	0.511^***^	0.42^***^	0.371^**^				
Mutual explained variance (ρ^2^)	0.2611	0.1764	0.1376				
Significance level test	0.000	0.000	0.008				

^**^p < 0.001, ^***^p < 0.001.

In this canonical correlation, among the variables in group X, two variables, including “organization and service” and “safety and convenience,” have absolute values of coefficients of the first canonical variable (ε_1_) that are all ≥0.30, which are 0.515 and 0.968, respectively. In the variables in group Y, eight variables including “funding input,” “facilities,” “organizational construction,” “backbone training,” “knowledge provision,” “publicity and mobilization,” “system construction,” and “monitoring service” have absolute values of coefficients in the first canonical variable (η_1_) that are all ≥0.30, which are −0.755, −0.798, 0.614, 0.514, 0.806, 0.504, −0.705, and −0.573, respectively. It shows that the main barriers for older adults to participate in physical exercise is “safety and convenience,” can have a significant negative correlation with “funding input,” “facilities,” “system construction,” and “monitoring service” in public sports services through the first canonical correlation (ε_1_ and η_1_), and a significant positive correlation with “organizational construction,” “backbone training,” “knowledge provision,” and “publicity and mobilization.”

(3) In the second canonical correlation, the canonical variable (ε_2_) derived from the variables in group X can explain 17.64% of the total variance of the canonical variable (η_2_) in the variables in group Y. Meanwhile, the second canonical variable (η_2_) of the variables in group Y can account for 30.77% of the total variance of the variables in group Y. Therefore, the variables in group X, through their second canonical variable (ε_2_), can explain 5.43% of the total variance of the variables in group Y. Similarly, according to this calculation method, the variables in group Y, *via* their second canonical variable (η_2_), can explain 5.06% of the total variance of the variables in group X.

In this canonical correlation, among the variables in group X, the absolute value of the coefficient of “social support” in ε_2_ is ≥0.30 (*R* = 0.976). In the variables in group Y, five variables, including “activity organization,” “activity guidance,” “knowledge provision,” “publicity and mobilization,” and “system construction,” have absolute values of coefficients in the second canonical variable (η_2_) that are all ≥0.30, which are 0.526, 0.503, 0.744, 0.694, and 0.601, respectively. It shows that older adults need more “social support” for physical exercise, and such “support” can be addressed by strengthening sports services in aspects, such as “activity organization,” “activity guidance,” “knowledge provision,” “publicity and mobilization,” and “system construction.”

(4) In the third canonical correlation, the canonical variable (ε_3_) derived from the variables in group X can explain 13.76% of the total variance of the canonical variable (η_3_) in the variables in group Y. Meanwhile, the third canonical variable (η_3_) of the variables in group Y can account for 21.65% of the total variance of the variables in group Y. Therefore, the variables in group X, through their third canonical variable (ε_3_), can explain 2.98% of the total variance of the variables in group Y. Similarly, according to this calculation method, the variables in group Y, *via* their third canonical variable (η_3_), can explain 2.41% of the total variance of the variables in group X.

In this canonical correlation, among the variables in group X, the absolute value of the coefficient of “organization and service” in ε_3_ is ≥0.30 (*R* = 0.875). In the variables in group Y, four variables, including “organizational construction,” “backbone training,” “activity organization,” and “activity guidance,” have absolute values of coefficients in the third canonical variable (η_3_) that are all ≥0.30, which are 0.801, 0.664, 0.701, and 0.760, respectively. It shows that “organization and service,” as an obstacle to older adults' physical exercise, can be addressed by strengthening “activity organization,” “activity guidance,” “knowledge provision,” “publicity and mobilization,” and “system construction” in public sports services.

(5) In the first canonical correlation (ε_1_ and η_1_), the variable that makes an important contribution to ε_1_ is “safety and convenience,” which mainly refers to the obstacles for older adults in physical exercise, such as safety and convenience. The variables that make important contributions to η_1_ are “knowledge provision” (0.806^**^), “facilities” (−0.798^**^), “funding input” (−0.755^**^), and “system construction” (−0.705^**^). This characteristic of the relationship is striking. It is entirely reasonable that “safety and convenience” in public sports services for older adults has a significant positive correlation with “knowledge provision.” However, it has a significant negative correlation with “facilities,” “funding input” and “system construction,” which is clearly illogical. This indicates that there are certain problems with the current use of public sports service funding for rural older adults. For example, there are safety hazards related to equipment installation and protection, and problems such as inconvenient fitness facilities and inadequate institutional construction, which increase the risks of exercise and reduce the efficiency and effectiveness of facility utilization.

In the second canonical correlation (ε_2_ and η_2_), the variable that makes an important contribution to ε_2_ is “social support,” which mainly refers to the need for support from family members and friends for older adults' participation in physical exercise. The variables that make important contributions to η_2_ are “knowledge provision” (0.744^**^) and “publicity and mobilization” (0.694^**^). The standardized coefficients of the variables contributing to both ε_2_ and η_2_ are in the same direction. This indicates that overcoming the obstacle of “social support” in older adults' physical exercise can be achieved through measures such as strengthening “knowledge provision,” enhancing the level of “publicity and mobilization,” improving “activity guidance,” and perfecting “system construction” in public sports services.

In the third canonical correlation (ε_3_ and η_3_), the variable that makes an important contribution to ε_3_ is “organization and service.” The obstacle of “organization and service” in older adults‘ physical exercise refers to the fact that their exercise needs guidance, planned and organized activities, especially the availability of relevant services on demand. The variables that make important contributions to η_3_ are “organizational construction” (0.801^**^), “activity guidance” (0.760^**^), and “activity organization” (0.701^**^), etc. The standardized coefficients of the variables contributing to both ε_3_ and η_3_ are in the same direction. This indicates that overcoming the obstacle of “organization and service” in older adults' physical exercise can be achieved by strengthening “organizational construction,” “activity guidance,” and “activity organization” in public sports services.

In addition, the percentage of overlapping variance shows that the canonical variables ε_1_, ε_2_, and ε_3_ in group X can explain 20.2% (11.79% + 5.43% + 2.98%) of the variance of the 10 variables in group Y, while the canonical variables η_1_, η_2_, and η_3_ in group Y can explain 17.95% (10.48% + 5.06% + 2.41%) of the three variables in group X. Obviously, the difference between the two percentages of overlapping variance is not significant, which indicates that the current obstacles encountered by rural older adults in physical exercise can basically be solved through the current supply-demand matching of public sports services.

#### 4.3.4 Correlation between rural older adults' intention, behavior, and effect of physical activity and the match between supply and demand of public sports services

**Figure 5:**
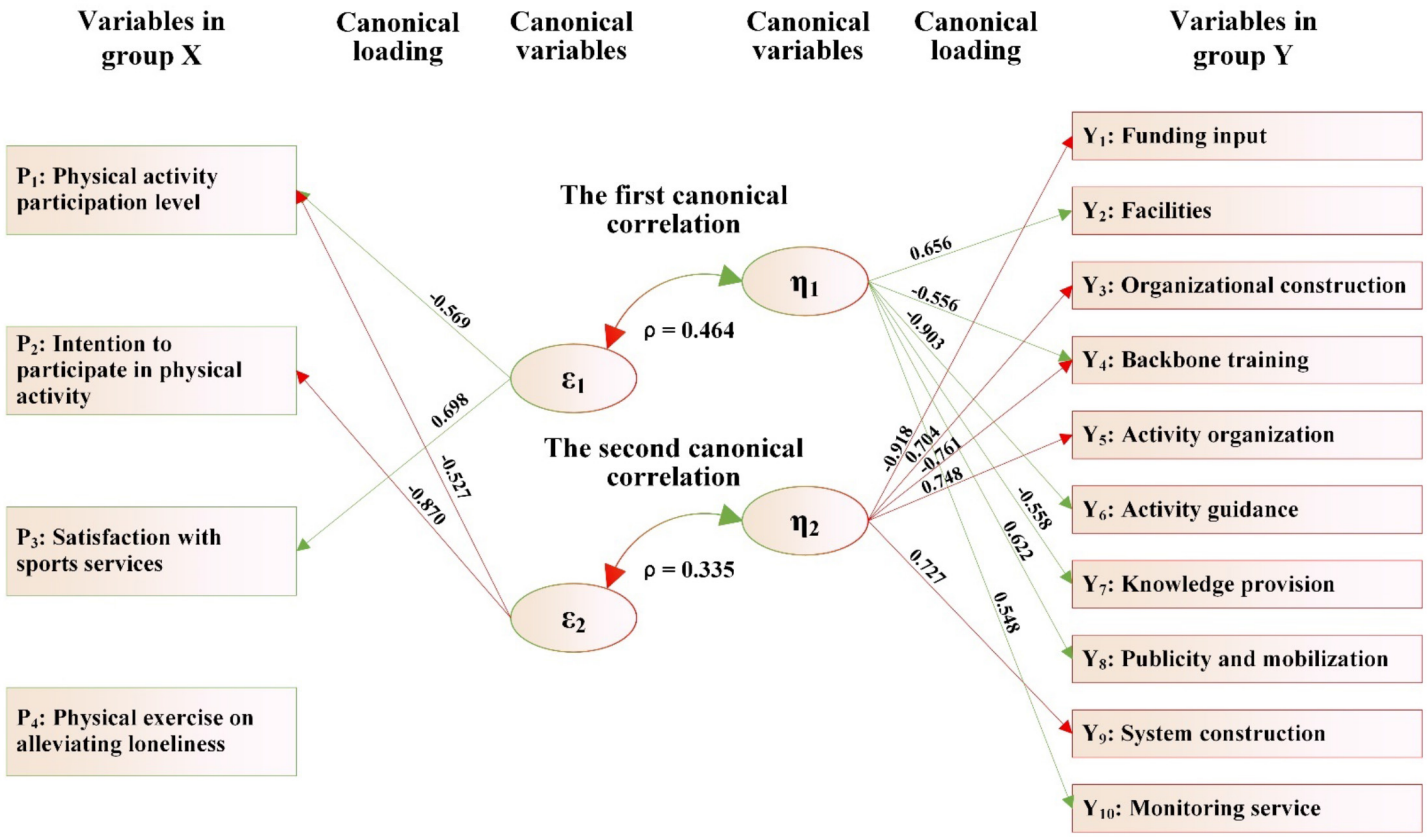
The canonical correlation path between rural older adults' intention, behavior, and effect of physical activity and the match between supply and demand of public sports services.

Figure 5, given in Table 14, can be found:

(1) The two canonical variables (ε_1_ and ε_2_) extracted from the variables in group X (including two dimensions: P_1_, P_2_, P_3_, and P_4_) and the two canonical variables (η_1_ and η_2_) extracted from the variables in group Y (Y_1_-Y_10_) form the first canonical correlation (ε_1_ and η_1_) and the second canonical correlation (ε_2_ and η_2_), respectively.
(2) In the first canonical correlation, the canonical correlation coefficient ρ = 0.464^***^, indicating that the canonical variable ε_1_ derived from group X can explain 21.53% of the total variance of the canonical variable η_1_ in group Y. Meanwhile, the first canonical variable (η_1_) of the variables in group Y can account for 57.57% of the total variance of the variables in group Y. Therefore, the variables in group X, through their first canonical variable (ε_1_), can explain 12.39% of the total variance of the variables in group Y. Similarly, according to this calculation method, the variables in group Y, *via* their first canonical variable (η_1_), can explain 8.72% of the total variance of the variables in group X.

**Table 14:** The canonical correlation analysis table between the behavior and effect of physical activity of older adults, and the matching between supply and demand of public sports services.

**Variables**	**Canonical variables**		**Variables**	**Canonical variables**	
**(X)**	ε_1_	ε_2_	**(Y)**	η_1_	η_2_
P_1_: Physical activity participation level	−0.569	−0.527	Y_1_: Funding input	−0.350	−0.918
P_2_: Intention to participate in physical activity	0.383	−0.870	Y_2_: Facilities	0.656	−0.128
P_3_: Satisfaction with sports services	0.698	0.005	Y_3_: Organizational construction	−0.335	0.704
P_4_: Physical exercise on alleviating loneliness	0.191	0.060	Y_4_: Backbone training	−0.556	−0.761
			Y_5_: Activity organization	0.288	0.748
			Y_6_: Activity guidance	−0.903	−0.214
			Y_7_: Knowledge provision	−0.558	0.104
			Y_8_: Publicity and mobilization	0.622	−0.196
			Y_9_: System construction	0.225	0.727
			Y_10_: Monitoring service	0.548	−0.235
Sampling variation (%)	40.51	25.64		57.57	27.46
Overlapping Variance (%)	8.72	2.88		12.39	3.08
Canonical correlation coefficient (ρ)	0.464^***^	0.335^***^			
Mutual explained variance (ρ^2^)	0.2153	0.1122			
Significance level test	0.000	0.000			

^*^p < 0.05, ^**^p < 0.01, ^***^p < 0.001.

In this canonical correlation, among the variables in group X, two variables, including “physical activity participation level” and “satisfaction with sports services,” have absolute values of coefficients of the first canonical variable (ε_1_) that are all ≥0.30, which are −0.569 and 0.698, respectively. In the variables in group Y, six variables including “facilities,” “backbone training,” “activity guidance,” “knowledge provision,” “publicity and mobilization,” and “monitoring service” have absolute values of coefficients in the first canonical variable (η_1_) that are all ≥0.30, which are 0.656, −0.556, −0.903, −0.558, 0.622, and 0.548, respectively. This shows that improving older adults' “satisfaction” and “participation level” in physical exercise is related to strengthening six aspects of public sports services, namely “facilities,” “backbone training,” “activity guidance,” “knowledge provision,” “publicity and mobilization,” and “monitoring service.”

(3) In the second canonical correlation, the canonical variable ε_2_ derived from the variables in group X can explain 11.22% of the total variance of the canonical variable η_2_ in the variables in group Y. Meanwhile, the second canonical variable (η_2_) of the variables in group Y can account for 27.46% of the total variance of the variables in group Y. Therefore, the variables in group X, through their second canonical variable (ε_2_), can explain 3.08% of the total variance of the variables in group Y. Similarly, according to this calculation method, the variables in group Y, *via* their second canonical variable (η_2_), can explain 2.88% of the total variance of the variables in group X.

In this canonical correlation, among the variables in group X, two variables, including “intention to participate in physical activity” and “satisfaction with sports services,” have absolute values of coefficients of the second canonical variable (ε_2_) that are all ≥0.30, which are −0.527 and −0.870, respectively. In the variables in group Y, four variables, including “funding input,” “organizational construction,” “backbone training,” and “activity organization,” have absolute values of coefficients in the second canonical variable (η_2_) that are all ≥0.30, which are −0.918, 0.704, −0.761, 0.748, and 0.727, respectively.

(4) In the first canonical correlation (ε_1_ and η_1_), the variables that make important contributions to ε_1_, “satisfaction with sports services” (0.698) shows an inverse correlation with the variables that make important contributions to η_1_, namely “activity guidance” (−0.903), “knowledge provision” (−0.558), and “backbone training” (−0.556). This indicates that there are problems in the supply-demand matching of “activity guidance,” “knowledge provision,” and “backbone training” in the current public sports services for rural older adults, resulting in low satisfaction among older adults in this regard. The variable that makes a secondary contribution to ε_1_ is the “physical activity participation level” (−0.569) of older adults, which shows an inverse correlation with the relatively important contributing variables “facilities” (0.656), “publicity and mobilization” (0.622), and “monitoring service” (0.548) in η_1_. This indicates that in the current rural public sports services for older adults, the supply-demand matching of “facilities,” “publicity and mobilization,” and “monitoring service” is not conducive to improving the current level of participation in physical exercise among rural older adults.

In the second canonical correlation (ε_2_ and η_2_), the variable that makes an important contribution to ε_2_ is “intention to participate in physical exercise” (−0.870), followed by “physical activity participation level” (−0.527). They show an inverse correlation with the important contributing variables “organizational development” (0.704), “activity organization” (0.748), and “system construction” (0.727) in η_2_. This indicates that in the current rural public sports services for older adults, the supply-demand matching of “organizational development,” “activity organization,” and “system construction” needs to be improved to ensure that they can truly enhance older adults' intention and intensity of participation in physical exercise.

In addition, the percentage of overlapping variance shows that the canonical variables ε_1_ and ε_2_ in group X can explain 15.47% (12.39% + 3.08%) of the 10 variables in group Y. The canonical variables η_1_ and η_2_ in group Y can explain 11.6% (8.72% + 2.88%) of the four variables in group X. Obviously, there is a significant difference between the two percentages of overlapping variance, which indicates that the current supply-demand matching of rural public sports services is still far from meeting the needs of older adults in terms of satisfaction, participation intention, and participation level in physical exercise.

## 5 Conclusion and recommendations

### 5.1 Conclusion

In the content system of 10 public sports services for rural older adults, including older adults backbone training for older adults, sports activities organization for older adults, and guidance for older adults to participate in sports activities, only the supply-demand relationship of the system construction of public sports services and physical fitness monitoring services for older adults are basically normal. Other public sports services fail to meet the basic matching standards, of which the value of “matching baseline” of sports funds investment, sports organization construction, guidance for participating in sports activities, and sports activity organization is even less than 50%. In public sports services with a normal supply-demand relationship, physical fitness monitoring services are often mistakenly regarded as medical examination services for older adults, so there are few physical examination services provided in rural areas in reality. In addition, the supply-demand relationship of the system construction of public sports services for older adults is normal, while other services fail to meet the basic matching standards, indicating that the execution of the system of public sports for older adults is insufficient. This shows the seriousness of the situation regarding the matching of supply and demand of public sports services for rural older adults in China. One reason for this is the lack of human, financial, and material resources for public sports services for rural older adults. As confirmed by the above research, service resources such as sports backbone, sports funds investment, and sports facilities for the rural older adults in China are facing a lack of problems. The chronic insufficiency in sports fund investment is a direct reflection of the limited fiscal capacity of local rural governments and a policy orientation that often prioritizes economic development over public welfare services. Another reason is the lack of a sound development model for public sports services for older adults. The fact that the “system construction” matching score is relatively high while the matching scores for actual services like “activity organization” and “guidance” are low points to a significant gap between policy-on-paper and on-the-ground implementation. This indicates a flawed execution mechanism, where formal systems exist but lack the dedicated personnel, funding, and administrative will to translate them into effective services for older adults.

The study also found that, based on the value of “matching baseline,” public sports services for older adults also showed the following characteristics: first, in the relatively developed villages, the value of “matching baseline” of public sports services is higher. Second, among all the surveyed sports services, the value of “matching baseline” for the same service theme content decreases in the eastern, western, and central regions in that order. This indicates that rural public sports services for older adults are closely related to the degree of attention paid by local governments and the state of economic and social development. At present, the matching of supply and demand for rural public sports services has basically met the needs of older adults in terms of the cognitive problems of physical exercise, and the obstacles to physical exercise encountered by rural older adults can basically be solved through the current matching of supply and demand for public sports services. However, the matching of supply and demand for rural public sports services is still unable to meet the needs of older adults in terms of their health conditions and lifestyles. Additionally, there is still a big gap between the satisfaction, intention and degree of participation of older adults in sports and exercise. There was still a big gap between “satisfaction,” “participation intention” and “participation degree” of physical exercise for older adults.

This study has some limitations. The demand-side sample, while representative of the general rural older adults population, was dominated by younger older adults (< 70 years old) and had a slight male majority. Consequently, the specific needs of the advanced-age older adults (>80 years old) and of rural women may be underrepresented. Future research should employ targeted stratified sampling to conduct more in-depth studies on these specific subgroups to ensure service provision can be tailored to their unique needs.

### 5.2 Suggestions

In order to promote the supply of public sports services for rural older adults, the following suggestions are made:

(1) At the strategic level, it is necessary to adhere to the goal orientation, unify the development ideas, clarify the strategic positioning and goals of public sports services for rural older adults, and focus on meeting the basic and urgent needs of rural older adults in the short term, while gradually improving the quality of services in the long term, and moving toward the direction of diversification and precision.
(2) At the planning level, it is necessary to strengthen the top-level design. This involves establishing a national-level coordinating body, such as an inter-ministerial committee led by the General Administration of Sport and involving the National Health Commission and the Ministry of Civil Affairs, to formulate special plans that clarify departmental responsibilities and ensure synergistic management.
(3) At the main body level, it is necessary to accelerate the transformation of government functions, reasonably divide the roles of the government and social organizations, and guide social forces to actively participate in sports services for rural older adults through the purchase of services by the government, policy support and other measures.
(4) At the content level, it is necessary to adhere to the demand orientation, establish a mechanism to express the demand for sports services among rural older adults, enhance the subjective awareness of older adults, and focus on the priority supply of sports organization services, guidance services and system construction, so as to form a diversified combination of service contents.
(5) At the mechanism level, it is necessary to improve the demand-oriented supply mechanism, establish a coordinated supply system for multiple subjects, promote government subsidies, contract outsourcing, volunteer services and other supply modes, and build a complete operation mechanism for demand expression, decision-making coordination, efficiency incentives and supervision and evaluation, so as to comprehensively improve the supply efficiency.
(6) At the technical level, it is necessary to build an integrated online-offline service platform. The government could lead this initiative through public-private partnerships, creating a unified national platform providing accessible exercise tutorials, information on local activities, and access to social sports instructors. Offline, existing community centers can be leveraged to provide tangible health services and guidance. This hybrid model, inspired by successful international cases like Japan's “Comprehensive Community Sports Clubs,” would effectively combine digital reach with grassroots implementation to enhance service capacity.

## Data Availability

The raw data supporting the conclusions of this article will be made available by the authors, without undue reservation.
